# Comparison of transcatheter aortic valve implantation with other approaches to treat aortic valve stenosis: a systematic review and meta-analysis

**DOI:** 10.1186/s13643-019-0954-3

**Published:** 2019-02-05

**Authors:** Gernot Wagner, Sabine Steiner, Gerald Gartlehner, Henrike Arfsten, Brigitte Wildner, Harald Mayr, Deddo Moertl

**Affiliations:** 1 Department for Evidence-based Medicine and Clinical Epidemiology, Danube University Krems, Dr.-Karl-Dorrek-Straße 30, 3500 Krems, Austria; 2 Division of Interventional Angiology, University Hospital Leipzig, Liebigstraße 20, Haus 4, 04103 Leipzig, Germany; 3 RTI International, 3040 East Cornwallis Road, PO Box 12194, Research Triangle Park, NC 27709-2194 USA; 4 Department of Medicine II, Division of Cardiology, Medical University of Vienna, Waehringer Guertel 18-20, 1090 Vienna, Austria; 5 University Library-Information Retrieval Office, Medical University of Vienna, Waehringer Guertel 18-20, 1090 Vienna, Austria; 6 Department of Internal Medicine 3, University Hospital St. Poelten, Karl Landsteiner University of Health Sciences, Dunant-Platz 1, 3100 St. Poelten, Austria; 7 Institute for Research of Ischaemic Cardiac Disease and Rhythmology, Karl Landsteiner Society, Dunant-Platz 1, 3100 St. Poelten, Austria

**Keywords:** Severe aortic stenosis, Transcatheter aortic valve implantation, Systematic literature review

## Abstract

**Background:**

Transcatheter aortic valve replacement (TAVI) is an alternative treatment for patients with symptomatic severe aortic stenosis ineligible for surgical aortic valve replacement (SAVR) or at increased perioperative risk. Due to continually emerging evidence, we performed a systematic review and meta-analysis comparing benefits and harms of TAVI, SAVR, medical therapy, and balloon aortic valvuloplasty.

**Methods:**

We searched MEDLINE, Embase, and Cochrane CENTRAL from 2002 to June 6, 2017. We dually screened abstracts and full-text articles for randomized controlled trials (RCTs) and propensity score-matched observational studies. Two investigators independently rated the risk of bias of included studies and determined the certainty of evidence using GRADE (Grading of Recommendations Assessment, Development and Evaluation). If data permitted, we performed meta-analyses using random- and fixed-effects models.

**Results:**

Out of 7755 citations, we included six RCTs (5862 patients) and 13 observational studies (6376 patients). In meta-analyses, patients treated with SAVR or TAVI had similar risks for mortality at 30 days (relative risk [RR] 1.05; 95% confidence interval [CI] 0.82 to 1.33) and 1 year (RR 1.02; 95% CI 0.93 to 1.13). TAVI had significantly lower risks for major bleeding but increased risks for major vascular complications, moderate or severe paravalvular aortic regurgitation, and new pacemaker implantation compared to SAVR. Comparing TAVI to medical therapy, mortality did not differ at 30 days but was significantly reduced at 1 year (RR 0.51; 95% CI 0.34 to 0.77).

**Conclusions:**

Given similar mortality risks but different patterns of adverse events, the choice between TAVI and SAVR remains an individual one.

**Electronic supplementary material:**

The online version of this article (10.1186/s13643-019-0954-3) contains supplementary material, which is available to authorized users.

## Background

The prevalence of severe aortic stenosis (AS) increases with age to a value of 3.4% in people 75 years or older. Approximately one million elderly patients in the European countries and 540,000 in North America suffer from symptomatic severe aortic stenosis. These numbers are expected to increase due to demographic changes [1]. Since severe symptomatic AS is associated with increased mortality, prognosis without treatment is poor [2].

While in the past, surgical aortic valve replacement (SAVR) has been the only recommended treatment of choice in patients with symptomatic severe AS, transcatheter aortic valve replacement (TAVI) has emerged as an alternative treatment option over the last 15 years [3]. Today, the European Society of Cardiology (ESC) guidelines recommend TAVI in patients with severe symptomatic AS who are considered inoperable [3]. These recommendations are mainly based on one randomized controlled trial (RCT), the Placement of Aortic Transcatheter Valves (PARTNER) trial arm B [4], where TAVI was compared to medical therapy in patients who were considered inoperable [4]. In regard to patients who are deemed operable but at increased surgical risk, the decision between TAVI and SAVR should be made according to assessment of the interdisciplinary Heart Team, based on individual risk factors, and patient characteristics [3].

With the ongoing uptake of TAVI worldwide, the amount of published data is constantly increasing. In addition to initial clinical trials in high-risk patients [5–7], recent RCTs for intermediate-risk patient populations have been published [8, 9] showing non-inferiority regarding a composite endpoint of all-cause death or disabling stroke for TAVI as compared to SAVR. Supplementary to data from RCTs comparing TAVI with SAVR, non-randomized trials and observational studies are adding further information. Prior systematic reviews in the field do not cover all recently published trials comparing TAVI to SAVR [10–12].

Therefore, the aim of this systematic review is to summarize the efficacy, effectiveness, and safety of TAVI in patients with symptomatic severe aortic stenosis compared to SAVR and non-surgical management comprising balloon aortic valvuloplasty (BAV) and medical therapy.

## Methods

We adhered to the Preferred Reporting Items for Systematic Reviews and Meta-analyses (PRISMA) [13] statement throughout this manuscript (PRISMA checklist see Additional file 1).

### Data sources and searches

An experienced medical information specialist (BW) searched the electronic databases MEDLINE, Embase, and the Cochrane Central Register of Controlled Trials via Ovid on January 27, 2017, with an additional update performed on June 6, 2017. In the literature search, we went back in time up to January 2002, when TAVI was performed for the first time. As search terms, we used free-text terms and Medical Subject Headings (MeSH) in order to identify relevant references. The search strategy for each database used is provided in Additional file 2. In addition, we searched ClinicalTrials.gov to detect unpublished studies. In order to identify publications not found by searches in electronic databases, we checked reference lists of included articles and relevant reviews and manually searched websites of selected cardiovascular journals.

### Inclusion and exclusion criteria

All studies comparing TAVI for the treatment of severe symptomatic aortic valve stenosis to other treatment strategies including SAVR, BAV, and medical therapy were eligible. We present study eligibility criteria in detail in Table 1.

**Table 1:** Eligibility criteria for relevant studies

	Eligibility criteria
Populations	• Adult patients with severe, symptomatic, native aortic valve stenosis • Any risk profile (high, intermediate, low)
Intervention	• Transcatheter aortic valve implantation (TAVI) for native aortic valve stenosis with: - Any commercial-used valve device - Any transvascular or transapical percutaneous approach - With or without concomitant percutaneous coronary intervention
Comparators	• Surgical aortic valve replacement - Any valve devices and surgical approaches (conventional, minimally invasive) - With or without concomitant intervention (coronary aortic bypass grafting) • Medical therapy (MT) • Balloon aortic valvuloplasty
Outcomes	• Efficacy and effectiveness - Mortality 30 days - Mortality 1 year • Safety - Stroke 30 days, 1 year - Transient ischemic attack 30 days - Myocardial infarction 30 days - Major bleeding 30 days - Major vascular complications 30 days - Severe or moderate paravalvular aortic regurgitation 30 days - New pacemaker implantation 30 day We included efficacy, effectiveness, and safety outcome reported as in-hospital, perioperative, or postoperative.
Timing	• Minimum follow-up duration of 30 days
Study designs	• Randomized controlled trials • Non-randomized controlled trials • Controlled cohort studies with propensity score-matching • For all eligible study designs, 100 patients or more in the TAVI arm We excluded case reports, case series, and any study without control group and fewer than 100 patients in the TAVI arm.
Publication type	• Publication reporting primary data We excluded publications not reporting primary data (narrative reviews, systematic reviews, and meta-analysis) as well as abstracts only, letters, and editorials.
Publication language	• English, German

### Study selection

Two reviewers (DM, GW) performed an independent initial screening of citations by title and abstract. If predefined study eligibility criteria were met or the abstract was inconclusive, full-text was obtained and assessed for relevance. Each step of the study selection process was pilot tested. Disagreements between reviewers were solved by consensus or by involvement of a third reviewer. In case a study was published in multiple publications, the most comprehensive publication was included.

### Data extraction

An electronic data abstraction form was used to obtain study and procedural characteristics, baseline characteristics of the patient population, and outcome parameters of interest. A second reviewer checked extracted data for accuracy and completeness. In case of uncertainty or inconsistency of published data as well as a potential overlap of study populations from different publications, we contacted the corresponding authors via e-mail for clarification. In the absence of reported intention-to-treat analyses, data from per-protocol or as-treated analyses were extracted and indicated in a footnote.

### Risk of bias assessment and certainty of evidence

Two reviewers assessed the risk of bias (HA, GW) of included studies. For risk of bias assessment of RCTs, we used the Cochrane risk of bias tool [14] and the Newcastle-Ottawa Scale (NOS) for observational studies [15]. The Grading of Recommendations Assessment, Development and Evaluation (GRADE) approach was applied to assess the certainty of evidence (very low, low, moderate, high) [16].

### Data synthesis and analysis

For 30-day and 1-year mortality, we calculated relative risk with 95% confidence intervals (CI). Fixed-effects (Mantel-Haenszel method) [17] and random-effects meta-analyses (DerSimonian and Laird method) [18] of relative risk estimates were employed. Since we anticipated clinical heterogeneity across studies, we reported results only from random-effects models in the text; forest plots also depict results from fixed-effects models. We conducted subgroup analysis for different study types and risk populations. Heterogeneity across trials was assessed by visual inspection of the forest plots and calculation of *I*^2^ statistics [19, 20]. We assessed potential publication bias with Egger’s tests and the visual interpretation of funnel plots. We used Stata 14.2 (Stata Corp, College Station, TX, USA) for all statistical analyses. A *p* value of < 0.05 was considered statistically significant.

## Results

### Study characteristics and risk of bias

**Fig. 1:**
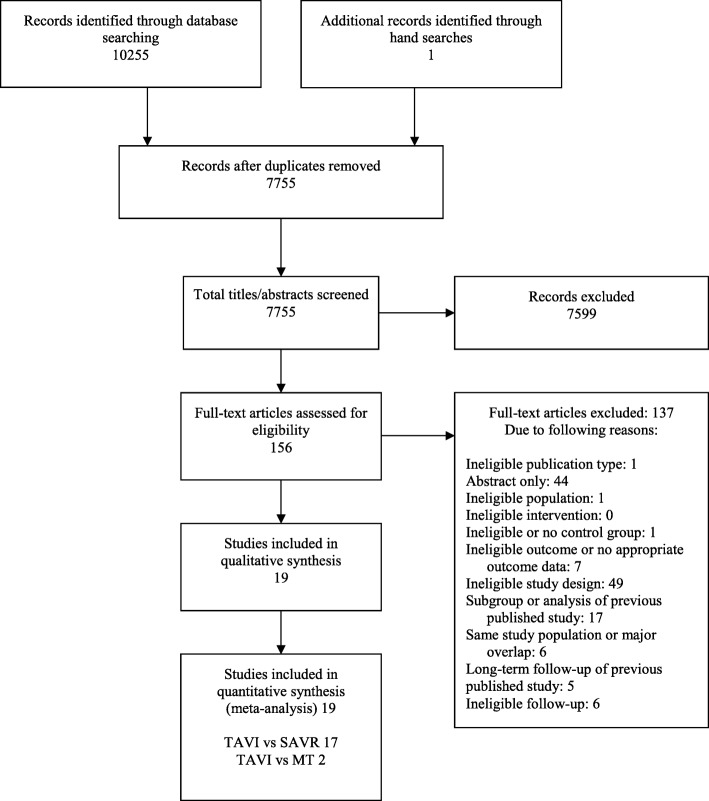
PRISMA flow diagram modified from Moher et al. [13] Abbreviations: MT = medical therapy, PRISMA *=* Preferred Reporting Items for Systematic Reviews and Meta-Analyses, SAVR = surgical aortic valve replacement, TAVI = transcatheter aortic valve replacement

After removal of duplicates, 7755 citations were screened by title and abstract. Subsequent full-text review included 19 studies with a total of 12,238 patients. Details of the study selection process are shown in the PRISMA flowchart (Fig. 1), and list of excluded full-text articles is provided in Additional file 2. Among the included six RCTs (5862 patients), five compared TAVI with SAVR [5, 7–9, 21], and one TAVI with medical therapy/BAV [4]. Furthermore, we included 13 observational propensity score-matched studies (6376 patients), of those 12 studies compared TAVI with SAVR [22–33] and one TAVI with medical therapy/BAV [34]. Based on our inclusion criteria, we did not identify any study comparing TAVI to BAV or medical therapy only.

The shortest follow-up period was 1 month [22, 33], whereas the longest was 5 years [21]. Most of the studies were conducted as multicenter studies [4, 5, 7–9, 21–24, 26, 28, 29]. Valve Academic Research Consortium (VARC), VARC-2, or VARC-like definitions were applied in several studies for all or certain clinical endpoints [4, 5, 7–9, 21–23, 27, 30, 31, 33]. Because of varying study durations and endpoints, the number of included studies in the respective meta-analyses varied.

Among the 19 selected studies, all six RCTs and 12 observational propensity score-matched studies were rated low risk of bias. Only one observational propensity score-matched study was rated as moderate risk of bias [24]. Detailed risk of bias assessments for all included studies are presented in Additional file 3.

### Patient and procedural characteristics

Mean age of study participants ranged from 74 to 85 years. The majority of studies included patients who were considered as high risk for surgery [4, 5, 7, 22–26, 32, 34]. However, recently published RCTs [8, 9, 21] and a few propensity score-matched observational studies [27–31, 33] investigated TAVI in intermediate- and low-risk patient populations. This is reflected by lower baseline risk scores (The European System for Cardiac Operative Risk Evaluation [euroSCORE] and the Society of Thoracic Surgeons [STS] score) of patients enrolled in those trials.

Overall, the majority of study participants reported *New York Heart Association* (NYHA) class III or IV at baseline. The mean left ventricular ejection fraction (LVEF) was greater than 50% in all included studies reporting LVEF. Although co-morbidities varied across studies, the prevalence was high in general. The percentage of patients with an implanted permanent pacemaker was more than 20% in three studies [4, 5, 7] and considerably lower or not reported in remaining studies [8, 9, 21–34] (Table A in Additional file 4). One study investigated only patients on dialysis [26]. Results of each individual study comparing TAVI with SAVR or medical therapy/BAV are summarized in Table B in Additional file 4.

In the included studies, both balloon-expanding (Edwards SAPIEN® and SAPIEN XT®) and/or self-expanding TAVI devices (Medtronic CoreValve® and Evolut R®, Symetis ACURATE®) were evaluated. Different access approaches were performed in most of the studies. Overall, the most common vascular access was transfemoral followed by transapical and other accesses such as transsubclavian or transaortic. In total, six studies exclusively applied one percutaneous approach for valve implantation (transfemoral [4, 27, 31], transapical [23, 25, 32]). Five out of six RCTs were funded by valve manufactures [4, 5, 7–9]. Detailed study characteristics are shown in Table 2.

**Table 2:** Characteristics of included studies

Study author and year	Study design	Risk of bias	Recruitment period	Study sites and country	Follow-up^a^(Months or years)	Intervention *N*^b^	TAVI device (%)^h^	TAVI access (%)^h^
Reardon 2017 [9] SURTAVI	RCT non-inferiority	Low	2012–2016	87 USA, Canada, and Europe	Max 2 years	TAVI 879 SAVR 867	Medtronic CoreValve (84) Medtronic Evolut R (16)	TF (NR) TS (NR) TAO (NR)
Leon 2016 [8] PARTNER 2A	RCT non-inferiority	Low	2011–2013	57 USA and Canada	Max 2 years	TAVI 1011 SAVR 1021	Edwards SAPIEN XT (100)	TF (76.7) TA (17.2) TAO (6.1)
Thyregod 2015 [21] NOTION	RCT superiority	Low	2009–2013	2 Denmark, 1 Sweden	Max 5 years	TAVI 145 SAVR 135	Medtronic CoreValve (100)	TF (96.5) TS (3.5)
Adams 2014 [7] US CoreValve	RCT non-inferiority and superiority	Low	2011–2012	45 USA	Mean 14.1 months 12.8 months	TAVI 394 SAVR 401	Medtronic CoreValve (100)	TF (82.8) TS and TAO (17.2)
Smith 2011 [5] PARTNER A	RCT non-inferiority	Low	2007–2009	22 USA, 2 Canada, 1 Germany	Median 1.4 years	TAVI 348 SAVR 351	Edwards SAPIEN (100)	TF (70.1) TA (29.9)
Leon 2010 [4] PARTNER B	RCT superiority	Low	2007–2009	21 (17 USA, 4 other)	Median 1.6 years	TAVI 179 MT 179 (150 MT + BAV)	Edwards SAPIEN (100)	TF (100)
Repossini 2017 [33]	Observational propensity matched	Low	2010–2014	7 EU	Max 1 month	TAVI 142 SAVR 142	Edwards SAPIEN XT (NR) Medtronic CoreValve (NR) Symetis ACURATE (NR)	TF (NR) TA (NR) Other transvascular (NR)
Hannan 2016 [24]	Observational propensity matched	Medium	2011–2012	17 USA	Max 1 year	TAVI 405 SAVR 405	NR	TF (84.7) TA (15.3)
D’Onofrio 2016 [23]	Observational propensity matched	Low	2007–2012	33 Italy	Max 1 year	TAVI 214 SAVR 214^e^	Edwards SAPIEN (NR) Edwards SAPIEN XT (NR)	TF (NR) TA (NR)
Kobrin 2015 [26]	Observational propensity matched	Low	2011–2012	Multicenter USA	Median 6.2 months^c^	TAVI 194 SAVR 194	NR	NR
Tamburino 2015 [31] OBSERVANT	Observational propensity matched	Low	2010–2012	93 Italy	Max 1 year	TAVI 650 SAVR 650	Edwards SAPIEN XT (44.9) Medtronic CoreValve (55.1)	TF (100)
Schymik 2015 [30]	Observational propensity matched	Low	2008–2012 2007–2012	1 Germany	Max 3 years	TAVI 216 SAVR 216	Edwards SAPIEN (NR) Edwards SAPIEN XT (NR) Medtronic CoreValve (NR) Symetis ACURATE (NR)	TF (NR) TA (NR)
Muneretto 2015^d^ [28]	Observational propensity matched	Low	2007–2014	7 Europe	Mean 2.7 years 4.4 years 2.3 years	TAVI 204 SAVR 408^f^	Edwards SAPIEN XT (38.7) Medtronic CoreValve (59.3) Symetis ACURATE (1.9)	TF (74.5) TA (24.5) Other transvessel approach (0.9)
Hoffmann 2013 [34]	Observational propensity matched	Low	2008–2009	1 Germany	Max 2 years	TAVI 135 MT 135 (13 MT + BAV)	Edwards SAPIEN (53.3) Medtronic CoreValve (46.7)	TA (53.3) TF (46.7)
D’Onofrio 2013 [22]	Observational propensity matched	Low	2008–2011 2009–2011	Multicenter Italy	Max 1 month	TAVI 143 SAVR 143^g^	Edwards SAPIEN (NR) Edwards SAPIEN XT (NR)	TA (100)
Piazza 2013 [29]	Observational propensity matched	Low	2006–2010	3 (Switzerland, Germany, the Netherlands)	Max 1 year	TAVI 405 SAVR 405	NR	NR
Latib 2012 [27]	Observational propensity matched	Low	2007–2011 2003–2008	1 Italy	Max 1 year	TAVI 111 SAVR 111	Edwards SAPIEN and SAPIEN XT (63.1) Medtronic CoreValve (36.9)	TF (100)
Holzhey 2012 [25]	Observational Propensity matched	Low	2006–2010 2001–2010	1 Germany	Mean 1.8 years	TAVI 167 SAVR 167	Edwards SAPIEN (100)	TA (100)
Walther 2010 [32]	Observational propensity matched	Low	2006–2008	1 Germany	Max 1 year	TAVI 100 SAVR 100	Edwards SAPIEN (100)	TA (100)

*BAV* balloon aortic valvuloplasty, *ITT* intention-to-treat population, *Max* maximum, *MT* medical therapy, *N* number of patients, *NR* not reported, *SAVR* surgical aortic valve replacement, *TAVI* transcatheter aortic valve replacement, *TA* transapical, *TAO* transaortic, *TF* transfemoral, *TS* transsubclavian, *USA* United States of America

^a^If mean or median follow-up is not available, maximum follow-up time was extracted

^b^ITT population

^c^TAVI patients

^d^Third treatment arm with sutureless surgical aortic valve replacement not extracted

^e^All patients received sutureless surgical aortic valve replacement

^f^204 patients received sutureless aortic valve replacement

^g^31 patients received sutureless aortic valve replacement

^h^Percentages refer to ITT or as-treated population

### Efficacy and effectiveness

#### TAVI compared to SAVR

**Fig. 2:**
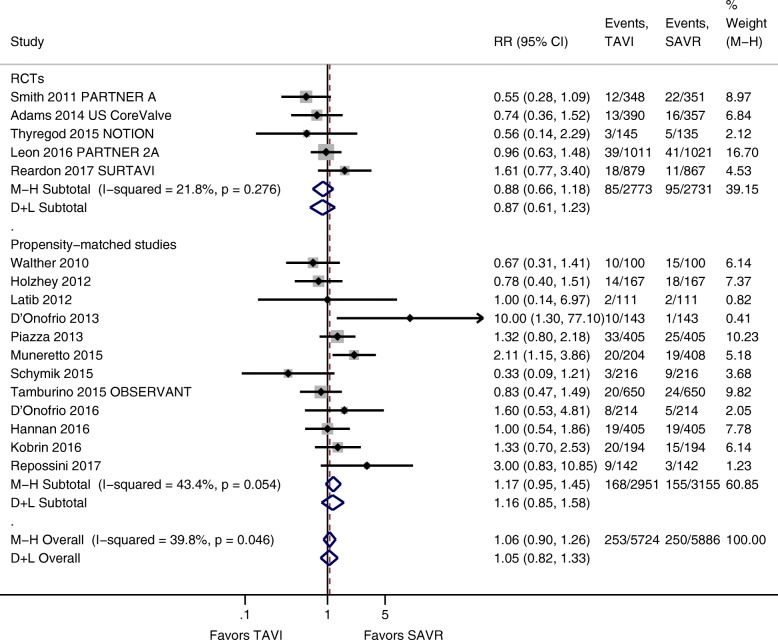
Forest plot 30-day mortality TAVI versus SAVR including randomized controlled trials and observational studies with propensity matching. Abbreviations: CI = confidence interval, D+L = DerSimonian and Laird method, M-H = Mantel-Haenszel method, NOTION = Nordic Aortic Valve Intervention, OBSERVANT = Observational Study of Effectiveness of SAVR-TAVR Procedures for Severe Aortic Stenosis Treatment, PARTNER = Placement of Aortic Transcatheter Valves, RCT = randomized controlled trial, RR = relative risk, SAVR = surgical aortic valve replacement, SURTAVI = Surgical Replacement and Transcatheter Aortic Valve Implantation, TAVI = transcatheter-aortic valve replacement, US = United States

Random-effects meta-analysis of 17 studies with a total number of 11,610 patients (5 RCTs, 12 propensity-matched studies) revealed no significant difference in 30-day mortality between TAVI and SAVR (4.4% versus 4.2%; RR 1.05; 95% CI 0.82 to 1.33; *I*^2^ = 39.8%; Fig. 2). Likewise, in subgroup analysis of RCTs (3.1% versus 3.5%; RR 0.87; 95% CI 0.61 to 1.23; *I*^2^ = 21.8%) and propensity score-matched observational studies (5.7% versus 4.9%; RR 1.16; 95% CI 0.85 to 1.58; *I*^2^ = 43.4%), no statistically significant difference could be noted.

**Fig. 3:**
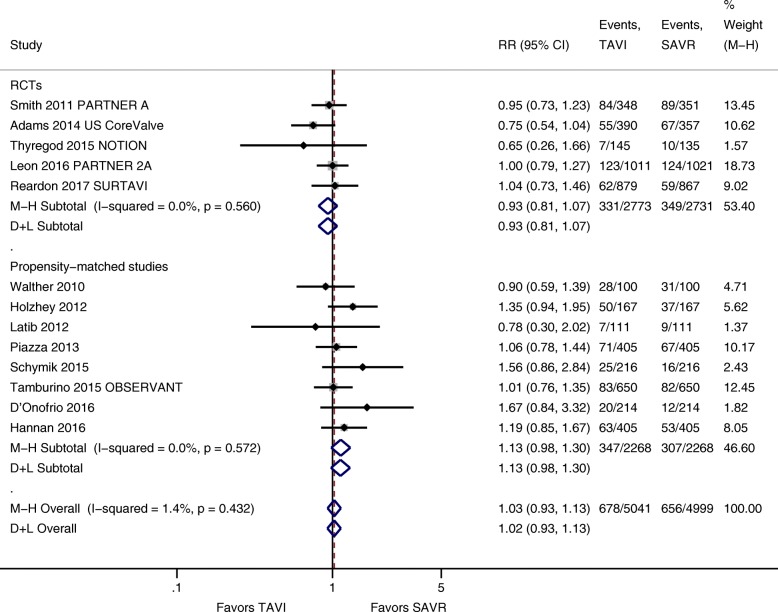
Forest-plot 1-year mortality TAVI versus SAVR including randomized controlled trials and observational studies with propensity matching. Abbreviations: CI = confidence interval, D+L = DerSimonian and Laird method, M-H = Mantel-Haenszel method, NOTION = Nordic Aortic Valve Intervention, OBSERVANT = Observational Study of Effectiveness of SAVR-TAVR Procedures for Severe Aortic Stenosis Treatment, PARTNER = Placement of Aortic Transcatheter Valves, RCT = randomized controlled trial, RR = relative risk, SAVR = surgical aortic valve replacement, SURTAVI = Surgical Replacement and Transcatheter Aortic Valve Implantation, TAVI = transcatheter-aortic valve replacement, US = United States

Regarding 1-year mortality, no significant difference was seen between TAVI and SAVR in a meta-analysis of 13 studies with a total number of 10,040 patients (5 RCTs, 8 propensity score-matched studies, 13.4% versus 13.1%; RR 1.02; 95% CI 0.93 to 1.13; *I*^2^ = 1.4%; Fig. 3). Both random-effects meta-analysis of RCTs (11.9% versus 12.8%; RR 0.93; 95% CI 0.81 to 1.07; *I*^2^ = 0%) and propensity score-matched observational studies (15.3% versus 13.5%; 1.13; 95% CI 0.98 to 1.30; *I*^2^ = 0%) showed no statistical significant difference between TAVI and SAVR. No statistically significant differences between RCTs and propensity score-matched studies could be identified.

Heterogeneity was moderate for 30-day (*I*^2^ = 39.8%) and negligible for 1-year mortality (*I*^2^ = 1.4%) (Figs. 2 and 3). Egger’s test and visual inspection of funnel plots did not suggest publication bias (see Additional file 5). Summary of findings tables with certainty of evidence rating is presented in Additional file 6.

Subgroup analysis of RCTs and propensity score-matched observational studies with high-risk patient populations yields similar all-cause mortality after TAVI and SAVR at 30 days (5.4% versus 5.7%; RR 0.92; 95% CI 0.65 to 1.31; *I*^*2*^ = 37.0%) and 1 year (18.5% versus 18.1%; RR 1.04; 95% CI 0.85 to 1.27; *I*^2^ = 44.1%). Random-effects meta-analysis of studies with intermediate- or lower-risk patients yields no statistically significant difference of those treated with TAVI compared to SAVR at 30 days (3.9% versus 3.5%; RR 1.17; 95% CI 0.84 to 1.63; *I*^2^ = 40.5%) and 1 year (11.1% versus 10.8%; RR 1.03; 95% CI 0.90 to 1.18; *I*^2^ = 0%) (Additional file 7).

#### TAVI compared with medical therapy

Only two studies (1 RCT, 1 propensity score-matched study) with a total of 628 patients compared TAVI with medical therapy, including 9.6% [34] and 83.8% [4] of patients with BAV in the medical therapy arm, respectively. A numerical trend for higher 30-day mortality with TAVI compared with medical therapy did not reach statistical significance, neither in the individual studies nor in random-effects meta-analysis (8.0% versus 4.8%; RR 1.66; 95% CI 0.90 to 3.08; *I*^2^ = 0%). In contrast, 1-year mortality was significantly reduced after TAVI (26.4% versus 50.3%; RR 0.51; 95% CI 0.34 to 0.77; *I*^2^ = 70%).

### Safety

#### TAVI compared to SAVR

A random-effects meta-analysis (15 studies, 9990 patients) yielded no statistically significant difference for risk of stroke at 30 days for patients treated with TAVI compared to SAVR (3.1% versus 3.6%; RR 0.84; 95% CI 0.64 to 1.10; *I*^2^ = 17.6%). Risk of stroke at 1 year was similar for patients treated with TAVI and SAVR (7 studies, 7035 patients, 6.3% versus 6.5%; RR 0.96; 95% CI 0.75 to 1.23; *I*^2^ = 34.8%).

Major bleeding at 30 days was statistically significantly lower for patients who underwent TAVI compared to SAVR, based on a random-effects meta-analysis (9 studies, 7198 patients, 13.0% versus 24.6%; RR 0.55; 95% CI 0.34 to 0.88; *I*^2^ = 94.2%). Based on a random-effects meta-analysis (10 studies, 8354 patients), risk of major vascular complications at 30 days was statistically significantly higher for patients treated with TAVI than with SAVR (7.8% versus 1.9%; RR 6.35; 95% CI 3.03 to 13.29; *I*^2^ = 79.6%). In addition, risk of moderate or severe paravalvular aortic regurgitation (8 studies, 6946 patients, 6.4% versus 0.9%; RR 6.86; 95% CI 4.71 to 9.99; *I*^2^ = 0%) and new pacemaker implantation (14 studies, 9790 patients, 14.4% versus 5.5%; RR 2.43; 95% CI 1.62 to 3.63; *I*^2^ = 84.0%) was statistically significantly higher for patients who underwent TAVI compared to SAVR.

**Fig. 4:**
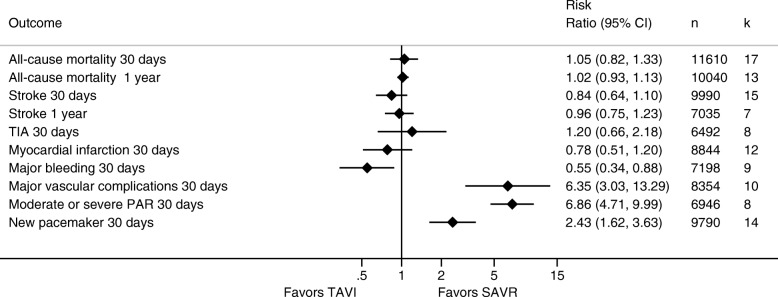
Results from random-effects meta-analysis comparing TAVI with SAVR and including randomized controlled trials and observational studies. Abbreviations: CI = confidence interval, k = number of studies, n = number of patients, PAR = paravalvular aortic regurgitation, SAVR = surgical aortic valve replacement, TAVI = transcatheter-aortic valve replacement, TIA = transient ischemic attack

>A random-effects meta-analysis yielded no statistically significant differences for risk of transient ischemic attack (TIA) (8 studies, 6492 patients, 1.0% versus 0.8%; RR 1.20; 95% CI 0.66 to 2.18; *I*^2^ = 12.6%) and myocardial infarction at 30 days (12 studies, 8844 patients, 0.9% versus 1.1%; RR 0.78; 95% CI 0.51 to 1.20; *I*^2^ = 0%). Results from meta-analysis for safety endpoints are presented in Fig. 4, and individual forest plots are provided in Additional file 8.

We provide rates of adverse events as well as endpoint definitions in individual studies in Additional file 4.

#### TAVI compared with medical therapy

In the multicenter RCT [4], the risk of stroke at 30 days was statistically significantly higher for patients treated with TAVI than medical therapy (6.7% versus 1.7%; RR 4.0; 95% CI 1.15 to 13.93). At 30 days, TAVI statistically significantly increased the risk of major bleeding, vascular complications, and moderate or severe paravalvular regurgitation. Within 30 days in this trial, no TIA and myocardial infarction occurred, neither in the TAVI nor the medical therapy group (Additional file 4).

## Discussion

TAVI has emerged as a promising alternative to SAVR, and increasing numbers of interventions are performed worldwide. This up-to-date systematic review and meta-analysis exhibited reassuring results for both methods as no difference was found with respect to all-cause mortality between TAVI and SAVR, both at 30 days and 1 year. Our results of comparable mortality between TAVI and SAVR at 30 days corroborate findings of other meta-analyses [10, 11, 35–37]. Similarly, the lack of difference in 1-year mortality between TAVI and SAVR is also consistent with results from previously published reviews [10, 36].

In order to gain information outside RCTs, we also included real-world observational data. While such studies can help to consolidate the evidence of an increasingly implemented intervention like TAVI, they are highly susceptible to bias and confounding. Thus, we limited these data to propensity score-matched studies of moderate size including at least 100 patients in the TAVI group.

The amount of evidence investigating the efficacy and safety of TAVI is constantly increasing. Importantly, this analysis also included recent trials such as PARTNER 2A [8] and SURTAVI [9], in which patients considered of intermediate perioperative risk have been included, reflecting the increasing use of TAVI not only in high-risk populations. While the less invasive nature of TAVI seems attractive, this comes at the price of higher numbers of adverse events, which are specifically related to the interventional nature of the procedure, such as major vascular complications, relevant paravalvular aortic regurgitation, and new pacemaker implantation. Further enhancement of implantation technique, operator skills, and valve prosthesis might reduce these events, but appropriate patient screening and selection will remain one of the most important factors. Due to the limitations of current risk scores in the setting of TAVI, additional interdisciplinary clinical judgment will continue to be crucial [38].

Importantly, SAVR was associated with more major bleeding than TAVI in this meta-analysis. However, as different bleeding classifications were used throughout the literature and standardized event adjudication is typically limited or absent in cohort studies, the extent of this difference is difficult to appraise and translate into individual patient information in clinical routine. Importantly, rates of cerebrovascular events and myocardial infarction were similar for both procedures, indicating no increased number of coronary events with open-heart surgery. Results from ongoing head-to-head comparisons of different TAVI approaches as well as long-term data on valve performance in large patient populations are needed to further clarify the value of TAVI for clinical routine.

We observed considerable heterogeneity (*I*^2^ > 50%) for major bleeding, major vascular complications, and new pacemaker implantation at 30 days. Application of different endpoint definitions (VARC, VARC-2, or others) might explain heterogeneity for bleeding and vascular complications. Different valve devices and generations for TAVI and SAVR contribute to heterogeneity regarding new pacemaker implantations due to different risks for impairment of the conducting system of the heart.

The higher mortality after 30 days is probably due to the invasive nature of TAVI compared with medical therapy, but then, there is a long-term benefit. TAVI reduces mortality at 1 year. Importantly, a relevant percentage of patients in the medical therapy groups also underwent BAV. Thus, this result underlines the current recommendation of the ESC guidelines on the management of valvular heart disease that BAV could be an option as a bridge to SAVR or TAVI in patients who are hemodynamically unstable or requiring urgent non-cardiac surgery [3].

Guidelines recommend the decision between TAVI and SAVR to be made by the Heart Team [3, 39]. Beyond risk scores associated with outcome data like mortality, the members of the Heart Team have to consider individual patient characteristics including frailty, impaired mobility, aortic sclerosis, chest deformation, and previous chest radiation, as well as comorbidities requiring additional interventions like mitral or tricuspid valve disease, coronary artery disease, and ascending aortic aneurysm [40]. Despite the variety of comorbidities complicating the decision for the best procedure, ongoing research has a strong focus on patients with low surgical risk or patients with moderate aortic stenosis and reduced left ventricular function in order to extend the indication for TAVI.

The following limitations of our systematic review have to be considered. First, despite propensity matching and similar baseline characteristics in the compared treatment groups, residual confounding might have been present in eligible observational studies. In particular, a certain degree of subjectivity remains in the decision of the Heart Team for or against performing a TAVI in an individual patient resulting from the nature of consensus opinions. Second, different patients’ risk profiles, valve devices, implantation techniques, and endpoint definitions might have contributed to heterogeneity. Third, a methodological limitation of this systematic review is the restriction to English and German publications. Finally, we have not registered or review in PROSPERO (International prospective register of systematic reviews). Like other systematic reviews, publication bias and selective outcome reporting are other potential limitations.

## Conclusion

This systematic review and meta-analysis summarizes the most recent evidence in the enhancing field of catheter-based treatment strategies for patients with symptomatic severe aortic stenosis. One-year mortality after TAVI was not significantly different than with SAVR but lower than with medical therapy. Considering that SAVR can be performed with acceptable clinical outcome even in high-surgical-risk patients with advanced age and comorbidities [41, 42], the decision between TAVI and SAVR currently remains an individual one in most patients. Beyond short- and long-term mortality, the Heart Team has to consider patients’ preferences, clinical characteristics, anatomical and technical aspects, and cardiac conditions requiring concomitant interventions for an informed decision on choice of treatment.

## Additional files

Additional file 1: PRISMA checklist. (DOCX 26 kb)

Additional file 2: Search strategies and excluded full-text articles. (DOCX 46 kb)

Additional file 3: Risk of bias assessment. (DOCX 22 kb)

Additional file 4: Patient characteristics and outcome in included studies. (DOCX 260 kb)

Additional file 5: Funnel plots and Egger’s test. (DOCX 37 kb)

Additional file 6: Summary of findings tables. (DOCX 24 kb)

Additional file 7: Subgroup analysis. (DOCX 35 kb)

Additional file 8: Forest-plots for safety endpoints TAVI versus SAVR. (DOCX 86 kb)

## Abbreviations

BAV: Balloon aortic valvuloplasty
CI: Confidence interval
euroSCORE: The European System for Cardiac Operative Risk Evaluation
GRADE: Grading of Recommendations Assessment, Development and Evaluation
LVEF: Left ventricular ejection fraction
MeSH: Medical Subject Headings
NOS: Newcastle-Ottawa Scale
*NYHA*: New York Heart Association
PRISMA: Preferred reporting items for systematic reviews and meta-analyses
RCT: Randomized controlled trial
RR: Relative risk
SAVR: Surgical aortic valve replacement
STS score: Society of Thoracic Surgeons score
TAVI: Transcatheter aortic valve implantation
