# Supplementary material for: Comparison of transcatheter aortic valve implantation with other approaches to treat aortic valve stenosis: a systematic review and meta-analysis
Source: Syst Rev. 2019 Feb 5;8:44. doi: 10.1186/s13643-019-0954-3 (PMC6362570; doi:10.1186/s13643-019-0954-3)
Supplement: Supplementary file 2 — Search strategies and excluded full-text articles. (DOCX 46 kb) [file 13643_2019_954_MOESM2_ESM.docx]

**Additional file 2. Search strategies and excluded full-text articles**

**Search strategies**

**MEDLINE (January 27, 2017)**

| # |  | Results |
| --- | --- | --- |
| 1 | exp Cardiac Catheterization/ | 52224 |
| 2 | exp Heart Valve Prosthesis/ | 33727 |
| 3 | exp Heart Valve Prosthesis Implantation/ | 20395 |
| 4 | 2 or 3 | 47098 |
| 5 | exp Aortic Valve/ | 29057 |
| 6 | exp Aortic Valve Stenosis/ | 38980 |
| 7 | 5 or 6 | 60683 |
| 8 | 1 and 4 and 7 | 3459 |
| 9 | ((transcutan* or transarterial* or percutan* or transcatheter* or transkatheter* or transapical* or transfemor* or transsubclav* or transaort* or trans- cutan* or trans-arterial* or trans-catheter* or trans-katheter* or trans-apical* or trans-femor* or trans-subclav* or trans-aort*) and aort* and valve* and (implant* or insert* or replace*)).ti. | 4715 |
| 10 | ((transcutan* or transarterial* or percutan* or transcatheter* or transkatheter* or transapical* or transfemor* or transsubclav* or transaort* or trans- cutan* or trans-arterial* or trans-catheter* or trans-katheter* or trans-apical* or trans-femor* or trans-subclav* or trans-aort*) and aort* and valve* and (implant* or insert* or replace*)).ab. | 5656 |
| 11 | (tavi or ta-tavi or tavr or pavi or pavr).ti. | 794 |
| 12 | (tavi or ta-tavi or tavr or pavi or pavr).ab. | 3330 |
| 13 | (aort* adj3 stenos*).ti,ab,sh,hw. | 29404 |
| 14 | (9 or 10 or 11 or 12) and 13 | 4884 |
| 15 | 8 or 14 | 6188 |
| 16 | randomized controlled trial.pt. | 509724 |
| 17 | controlled clinical trial.pt. | 98335 |
| 18 | (randomized controlled trial or randomized controlled trials as topic).sh. | 629268 |
| 19 | random allocation.sh. | 98755 |
| 20 | double-blind method.sh. | 158590 |
| 21 | single-blind method.sh. | 26711 |
| 22 | clinical trial.pt. | 548869 |
| 23 | exp Clinical Trial/ | 876377 |
| 24 | (clin$ adj5 trial$).ti. | 66755 |
| 25 | (clin$ adj5 trial$).ab. | 323987 |
| 26 | ((singl$ or doubl$ or trebl$ or tripl$) adj5 (blind$ or mask$)).ti. | 41429 |
| 27 | ((singl$ or doubl$ or trebl$ or tripl$) adj5 (blind$ or mask$)).ab. | 155110 |
| 28 | placebos.sh. | 37138 |
| 29 | placebo$.ti. | 34812 |
| 30 | placebo$.ab. | 203474 |
| 31 | random$.ti. | 191290 |
| 32 | random$.ab. | 977807 |
| 33 | research design.sh. | 105829 |
| 34 | comparative study.pt. | 1963863 |
| 35 | exp Evaluation Studies/ | 248668 |
| 36 | follow up studies.sh. | 631815 |
| 37 | prospective studies.sh. | 495160 |
| 38 | (control$ or rospective$ or volunteer$).ti. | 625008 |
| 39 | (control$ or rospective$ or volunteer$).ab. | 3844955 |
| 40 | 16 or 17 or 18 or 19 or 20 or 21 or 22 or 23 or 24 or 25 or 26 or 27 or 28 or 29 or 30 or 31 or 32 or 33 or 34 or 35 or 36 or 37 or 38 or 39 or exp Cohort Studies/ | 7546639 |
| 41 | exp Humans/ | 18314366 |
| 42 | exp Animals/ | 23175537 |
| 43 | 42 not (41 and 42) | 4861171 |
| 44 | 40 not 43 | 6224841 |
| 45 | 15 and 44 | 2575 |
| 46 | ((transcutan* or transarterial* or percutan* or transcatheter* or transkatheter* or transapical* or transfemor* or transsubclav* or transaort* or trans- cutan* or trans-arterial* or trans-catheter* or trans-katheter* or trans-apical* or trans-femor* or trans-subclav* or trans-aort*) and aort* and valve* and (implant* or insert* or replace*)).mp. | 7833 |
| 47 | (tavi or ta-tavi or tavr or pavi or pavr).mp. | 3939 |
| 48 | 46 or 47 | 8138 |
| 49 | meta-analysis.mp. or exp Meta-Analysis/ | 137211 |
| 50 | 48 and 49 | 149 |
| 51 | (surg* or operat*).ti. | 707100 |
| 52 | (ballon* or valvuloplast*).mp. [mp=title, abstract, original title, name of substance word, subject heading word, keyword heading word, protocol supplementary concept word, rare disease supplementary concept word, unique identifier] | 6280 |
| 53 | medical treatment.mp. | 42202 |
| 54 | exp Aortic Valve Stenosis/dt, th [Drug Therapy, Therapy] | 5846 |
| 55 | standard therapy.mp. [mp=title, abstract, original title, name of substance word, subject heading word, keyword heading word, protocol supplementary concept word, rare disease supplementary concept word, unique identifier] | 11547 |
| 56 | exp Treatment Outcome/ and (compar* or versus or vs).mp. [mp=title, abstract, original title, name of substance word, subject heading word, keyword heading word, protocol supplementary concept word, rare disease supplementary concept word, unique identifier] | 371011 |
| 57 | 51 or 52 or 53 or 54 or 55 or 56 | 1099617 |
| 58 | 50 and 57 | 65 |
| 59 | 45 or 58 | 2605 |
| 60 | limit 59 to yr=”2002 – 2017” | 2369 |
| 61 | limit 60 to yr=”2014 – 2017” | 1205 |

**Embase (January 27, 2017)**

| # |  | Results |
| --- | --- | --- |
| 1 | 'heart catheterization'/exp | 56,930 |
| 2 | 'heart valve replacement'/exp | 47,079 |
| 3 | 'heart valve prosthesis'/exp | 33,559 |
| 4 | 1 AND (2 OR 3) | 2,943 |
| 5 | 'transcatheter aortic valve implantation'/exp | 11,467 |
| 6 | 'aorta stenosis'/exp | 34,297 |
| 7 | 'aorta valve'/exp | 21,145 |
| 8 | 6 OR 7 | 49,647 |
| 9 | 4 OR 5 | 14,052 |
| 10 | 8 AND 9 | 7,803 |
| 11 | transcutan*:ti OR transarterial*:ti OR percutan*:ti OR transcatheter*:ti OR transkatheter*:ti OR transapical*:ti OR transfemor*:ti OR transsubclav*:ti OR transaort*:ti OR 'trans cutan*':ti OR 'trans arterial*':ti OR 'trans catheter*':ti OR 'trans katheter*':ti OR 'trans apical*':ti OR 'trans femor*':ti OR 'trans subclav*':ti OR 'trans aort*':ti AND aort*:ti AND valve*:ti AND (implant*:ti OR insert*:ti  OR replace*:ti) | 7,950 |
| 14 | tavi:ab OR 'ta tavi':ab OR tavr:ab OR pavi:ab OR pavr:ab  katheter*':ab OR 'trans apical*':ab OR 'trans femor*':ab OR 'trans subclav*':ab OR 'trans aort*':ab AND aort*:ab AND valve*:ab AND (implant*:ab OR insert*:ab OR replace*:ab) | 8,554 |
| 15 | (aort* NEAR/3 stenos*):ti,ab,de | 39,282 |
| 16 | 11 OR 12 OR 13 OR 14 | 13,659 |
| 17 | 15 AND 16 | 7,851 |
| 18 | 10 OR 17 | 9,792 |
| 19 | 'randomized controlled trial'/de | 429,350 |
| 20 | 'randomization'/exp | 72,005 |
| 21 | 'controlled study'/de | 5,223,575 |
| 22 | 'multicenter study'/de | 144,734 |
| 23 | 'phase 3 clinical trial'/de | 25,844 |
| 24 | 'phase 4 clinical trial'/de | 2,317 |
| 25 | 'double blind procedure'/de | 134,410 |
| 26 | 'single blind procedure'/de | 25,201 |
| 27 | random*:ti,ab OR crossover*:ti,ab OR 'cross over*':ti,ab OR factorial*:ti,ab OR placebo*:ti,ab OR volunteer*:ti,ab | 1,459,410 |
| 28 | ((singl* OR doubl* OR trebl* OR tripl*) NEAR/5 (blind* OR mask*)):ab,ti | 197,893 |
| 29 | 'animal'/de | 1,742,202 |
| 30 | 'nonhuman'/de | 4,952,739 |
| 31 | 'human'/de | 17,665,061 |
| 32 | 29 OR 30 NOT (31 AND (29 OR 30)) | 5,161,937 |
| 33 | 'major clinical study'/exp | 2,671,085 |
| 34 | 'controlled study'/exp | 5,469,895 |
| 35 | 'clinical article'/exp | 1,637,442 |
| 36 | random* OR trial* OR control* OR study OR compar* OR placebo* OR blind* OR doubl* | 16,765,308 |
| 37 | 'cohort analysis'/exp | 268,475 |
| 38 | 19 OR 20 OR 21 OR 22 OR 23 OR 24 OR 25 OR 26 OR 27 OR 28 OR 33 OR 34 OR 35 OR 36 OR 37 | 17,170,409 |
| 39 | 38 NOT 32 | 13,738,079 |
| 40 | 18 AND 39 | 6,388 |
| 41 | 40 AND (2002:py OR 2003:py OR 2004:py OR 2005:py OR 2006:py OR 2007:py OR 2008:py OR 2009:py OR 2010:py OR 2011:py OR 2012:py OR 2013:py OR 2014:py OR 2015:py OR 2016:py OR 2017:py) | 6,293 |
| 42 | 41 AND [13-1-2015]/sd NOT [20-1-2017]/sd | 2,333 |
| 43 | tavi:ab OR 'ta tavi':ab OR tavr:ab OR pavi:ab OR pavr | 8,556 |
| 44 | transcutan* OR transarterial* OR percutan* OR transcatheter* OR transkatheter* OR transapical* OR transfemor* OR transsubclav* OR transaort* OR 'trans cutan*' OR 'trans arterial*' OR 'trans catheter*' OR 'trans katheter*' OR 'trans apical*' OR 'trans femor*' OR 'trans subclav*' OR 'trans aort*' AND aort* AND valve* AND (implant* OR insert* OR replace*) | 16,376 |
| 45 | 43 OR 44 | 16,609 |
| 46 | 'meta analysis' OR 'meta-analysis'/exp | 179,280 |
| 47 | 45 AND 46 | 355 |
| 48 | surg*:ti OR operat*:ti | 799,683 |
| 49 | ballon* OR valvuloplast* | 13,167 |
| 50 | 'medical treatment' | 55,940 |
| 51 | 'aorta stenosis'/exp/dm_dt,dm_th | 2,731 |
| 52 | 'aorta valve stenosis'/dm_dt,dm_th | 1,246 |
| 53 | 'standard therapy' | 16,563 |
| 54 | 'treatment outcome'/exp AND (compar* OR versus OR vs) | 462,084 |
| 55 | 48 OR 49 OR 50 OR 51 OR 52 OR 53 OR 54 | 1,300,052 |
| 56 | 47 AND 55 | 138 |
| 57 | 40 OR 56 | 6,500 |
| 58 | 57 AND (2002:py OR 2003:py OR 2004:py OR 2005:py OR 2006:py OR 2007:py OR 2008:py OR 2009:py OR 2010:py OR 2011:py OR 2012:py OR 2013:py OR 2014:py OR 2015:py OR 2016:py OR 2017:py) | 6,340 |
| 59 | 57 AND (2002:py OR 2003:py OR 2004:py OR 2005:py OR 2006:py OR 2007:py OR 2008:py OR 2009:py OR 2010:py OR 2011:py OR 2012:py OR 2013:py OR 2014:py OR 2015:py OR 2016:py OR 2017:py) AND [14-1-2015]/sd NOT [27-1-2017]/sd | 2,434 |

**Cochrane Central Register of Controlled Trials (January 27, 2017)**

| # |  | Results |
| --- | --- | --- |
| 1 | exp Cardiac Catheterization/ | 2224 |
| 2 | exp Heart Valve Prosthesis/ | 460 |
| 3 | exp Heart Valve Prosthesis Implantation/ | 519 |
| 4 | 2 or 3 | 863 |
| 5 | exp Aortic Valve/ | 350 |
| 6 | exp Aortic Valve Stenosis/ | 486 |
| 7 | 5 or 6 | 722 |
| 8 | 1 and 4 and 7 | 68 |
| 9 | ((transcutan* or transarterial* or percutan* or transcatheter* or transkatheter* or transapical* or transfemor* or transsubclav* or transaort* or trans-cutan* or trans-arterial* or trans-catheter* or trans-katheter* or trans-apical* or trans-femor* or trans-subclav* or trans-aort*) and aort* and valve* and (implant* or insert* or replace*)).ti. | 252 |
| 10 | ((transcutan* or transarterial* or percutan* or transcatheter* or transkatheter* or transapical* or transfemor* or transsubclav* or transaort* or trans-cutan* or trans-arterial* or trans-catheter* or trans-katheter* or trans-apical* or trans-femor* or trans-subclav* or trans-aort*) and aort* and valve* and (implant* or insert* or replace*)).ab. | 309 |
| 11 | (tavi or ta-tavi or tavr or pavi or pavr).ti. | 49 |
| 12 | (tavi or ta-tavi or tavr or pavi or pavr).ab. | 294 |
| 13 | (aort* adj3 stenos*).ti,ab,sh,hw. | 705 |
| 14 | (9 or 10 or 11 or 12) and 13 | 263 |
| 15 | 8 or 14 | 275 |
| 16 | ((transcutan* or transarterial* or percutan* or transcatheter* or transkatheter* or transapical* or transfemor* or transsubclav* or transaort* or trans-cutan* or trans-arterial* or trans-catheter* or trans-katheter* or trans-apical* or trans-femor* or trans-subclav* or trans-aort*) and aort* and valve* and (implant* or insert* or replace*)).mp. | 360 |
| 17 | (tavi or ta-tavi or tavr or pavi or pavr).mp. | 299 |
| 18 | 16 or 17 | 376 |
| 19 | meta-analysis.mp. or exp Meta-Analysis/ | 3884 |
| 20 | 18 and 19 | 9 |
| 21 | (surg* or operat*).ti. | 43397 |
| 22 | (ballon* or valvuloplast*).mp. [mp=title, original title, abstract, mesh headings, heading words, keyword] | 205 |
| 23 | medical treatment.mp. | 2735 |
| 24 | exp Aortic Valve Stenosis/dt, th [Drug Therapy, Therapy] | 147 |
| 25 | standard therapy.mp. [mp=title, original title, abstract, mesh headings, heading words, keyword] | 2557 |
| 26 | exp Treatment Outcome/ and (compar* or versus or vs).mp. [mp=title, original title, abstract, mesh headings, heading words, keyword] | 74278 |
| 27 | 21 or 22 or 23 or 24 or 25 or 26 | 117702 |
| 28 | 20 and 27 | 2 |
| 29 | 15 or 28 | 275 |
| 30 | limit 29 to yr="2002 - 2017" | 269 |
| 31 | limit 30 to yr="2014 - 2017" | 179 |

**MEDLINE (June 6, 2017)**

| # |  | Results |
| --- | --- | --- |
| 1 | exp Cardiac Catheterization/ | 47502 |
| 2 | exp Heart Valve Prosthesis/ | 32082 |
| 3 | exp Heart Valve Prosthesis Implantation/ | 19897 |
| 4 | 2 or 3 | 45093 |
| 5 | exp Aortic Valve/ | 26986 |
| 6 | exp Aortic Valve Stenosis/ | 36300 |
| 7 | 5 or 6 | 56291 |
| 8 | 1 and 4 and 7 | 3298 |
| 9 | ((transcutan* or transarterial* or percutan* or transcatheter* or transkatheter* or transapical* or transfemor* or transsubclav* or transaort* or trans-cutan* or trans-arterial* or trans-catheter* or trans-katheter* or trans-apical* or trans-femor* or trans-subclav* or trans-aort*) and aort* and valve* and (implant* or insert* or replace*)).ti. | 4657 |
| 10 | ((transcutan* or transarterial* or percutan* or transcatheter* or transkatheter* or transapical* or transfemor* or transsubclav* or transaort* or trans-cutan* or trans-arterial* or trans-catheter* or trans-katheter* or trans-apical* or trans-femor* or trans-subclav* or trans-aort*) and aort* and valve* and (implant* or insert* or replace*)).ab. | 5655 |
| 11 | (tavi or ta-tavi or tavr or pavi or pavr).ti. | 779 |
| 12 | (tavi or ta-tavi or tavr or pavi or pavr).ab. | 3419 |
| 13 | (aort* adj3 stenos*).ti,ab,sh,hw. | 27742 |
| 14 | (9 or 10 or 11 or 12) and 13 | 4899 |
| 15 | 8 or 14 | 6094 |
| 16 | randomized controlled trial.pt. | 464676 |
| 17 | controlled clinical trial.pt. | 94172 |
| 18 | (randomized controlled trial or randomized controlled trials as topic).sh. | 572767 |
| 19 | random allocation.sh. | 92938 |
| 20 | double-blind method.sh. | 147591 |
| 21 | single-blind method.sh. | 24687 |
| 22 | clinical trial.pt. | 522007 |
| 23 | exp Clinical Trial/ | 806332 |
| 24 | (clin$ adj5 trial$).ti. | 61290 |
| 25 | (clin$ adj5 trial$).ab. | 290804 |
| 26 | ((singl$ or doubl$ or trebl$ or tripl$) adj5 (blind$ or mask$)).ti. | 39383 |
| 27 | ((singl$ or doubl$ or trebl$ or tripl$) adj5 (blind$ or mask$)).ab. | 145536 |
| 28 | placebos.sh. | 35003 |
| 29 | placebo$.ti. | 32569 |
| 30 | placebo$.ab. | 188031 |
| 31 | random$.ti. | 175203 |
| 32 | random$.ab. | 907998 |
| 33 | research design.sh. | 96379 |
| 34 | comparative study.pt. | 1813523 |
| 35 | exp Evaluation Studies/ | 232967 |
| 36 | follow up studies.sh. | 588527 |
| 37 | prospective studies.sh. | 459688 |
| 38 | (control$ or prospectiv$ or volunteer$).ti. | 576583 |
| 39 | (control$ or prospectiv$ or volunteer$).ab. | 3550548 |
| 40 | 16 or 17 or 18 or 19 or 20 or 21 or 22 or 23 or 24 or 25 or 26 or 27 or 28 or 29 or 30 or 31 or 32 or 33 or 34 or 35 or 36 or 37 or 38 or 39 or exp Cohort Studies/ | 6985629 |
| 41 | exp Humans/ | 16919841 |
| 42 | exp Animals/ | 21331512 |
| 43 | 42 not (41 and 42) | 4411671 |
| 44 | 40 not 43 | 5789652 |
| 45 | 15 and 44 | 2595 |
| 46 | ((transcutan* or transarterial* or percutan* or transcatheter* or transkatheter* or transapical* or transfemor* or transsubclav* or transaort* or trans-cutan* or trans-arterial* or trans-catheter* or trans-katheter* or trans-apical* or trans-femor* or trans-subclav* or trans-aort*) and aort* and valve* and (implant* or insert* or replace*)).mp. | 7781 |
| 47 | (tavi or ta-tavi or tavr or pavi or pavr).mp. | 3999 |
| 48 | 46 or 47 | 8069 |
| 49 | meta-analysis.mp. or exp Meta-Analysis/ | 128265 |
| 50 | 48 and 49 | 165 |
| 51 | (surg* or operat*).ti. | 670754 |
| 52 | (ballon* or valvuloplast*).mp. [mp=title, abstract, original title, name of substance word, subject heading word, keyword heading word, protocol supplementary concept word, rare disease supplementary concept word, unique identifier, synonyms] | 5967 |
| 53 | medical treatment.mp. | 40584 |
| 54 | exp Aortic Valve Stenosis/dt, th [Drug Therapy, Therapy] | 5514 |
| 55 | standard therapy.mp. [mp=title, abstract, original title, name of substance word, subject heading word, keyword heading word, protocol supplementary concept word, rare disease supplementary concept word, unique identifier, synonyms] | 10782 |
| 56 | exp Treatment Outcome/ and (compar* or versus or vs).mp. [mp=title, abstract, original title, name of substance word, subject heading word, keyword heading word, protocol supplementary concept word, rare disease supplementary concept word, unique identifier, synonyms] | 346731 |
| 57 | 51 or 52 or 53 or 54 or 55 or 56 | 1037748 |
| 58 | 50 and 57 | 78 |
| 59 | 45 or 58 | 2631 |
| 60 | limit 59 to yr="2002 -Current" | 2413 |
| 61 | limit 60 to yr="2016 -Current" | 515 |

**Embase (June 6, 2017)**

| # |  | Results |
| --- | --- | --- |
| 1 | 'heart catheterization'/exp | 58,025 |
| 2 | 'heart valve replacement'/exp | 48,919 |
| 3 | 'heart valve prosthesis'/exp | 34,426 |
| 4 | 1 AND (2 OR 3) | 3,037 |
| 5 | 'transcatheter aortic valve implantation'/exp | 12,354 |
| 6 | 'aorta stenosis'/exp | 35,298 |
| 7 | 'aorta valve'/exp | 21,500 |
| 8 | 6 OR 7 | 50,908 |
| 9 | 4 OR 5 | 15,000 |
| 10 | 8 AND 9 | 8,245 |
| 11 | transcutan*:ti OR transarterial*:ti OR percutan*:ti OR transcatheter*:ti OR transkatheter*:ti OR transapical*:ti OR transfemor*:ti OR transsubclav*:ti OR transaort*:ti OR 'trans cutan*':ti OR 'trans arterial*':ti OR 'trans catheter*':ti OR 'trans katheter*':ti OR 'trans apical*':ti OR 'trans femor*':ti OR 'trans subclav*':ti OR 'trans aort*':ti AND aort*:ti AND valve*:ti AND (implant*:ti OR insert*:ti OR replace*:ti) | 8,496 |
| 12 | transcutan*:ab OR transarterial*:ab OR percutan*:ab OR transcatheter*:ab OR transkatheter*:ab OR transapical*:ab OR transfemor*:ab OR transsubclav*:ab OR transaort*:ab OR 'trans cutan*':ab OR 'trans arterial*':ab OR 'trans catheter*':ab OR 'trans katheter*':ab OR 'trans apical*':ab OR 'trans femor*':ab OR 'trans subclav*':ab OR 'trans aort*':ab AND aort*:ab AND valve*:ab AND (implant*:ab OR insert*:ab OR replace*:ab) | 11,748 |
| 13 | tavi:ti OR 'ta tavi':ti OR tavr:ti OR pavi:ti OR pavr:ti | 2,519 |
| 14 | tavi:ab OR 'ta tavi':ab OR tavr:ab OR pavi:ab OR pavr:ab | 8,998 |
| 15 | (aort* NEAR/3 stenos*):ti,ab,de | 40,856 |
| 16 | 11 OR 12 OR 13 OR 14 | 14,575 |
| 17 | 15 AND 16 | 8,379 |
| 18 | 10 OR 17 | 10,406 |
| 19 | 'randomized controlled trial'/de | 447,991 |
| 20 | 'randomization'/exp | 73,693 |
| 21 | 'controlled study'/de | 5,417,607 |
| 22 | 'multicenter study'/de | 153,532 |
| 23 | 'phase 3 clinical trial'/de | 27,631 |
| 24 | 'phase 4 clinical trial'/de | 2,504 |
| 25 | 'double blind procedure'/de | 137,917 |
| 26 | 'single blind procedure'/de | 27,124 |
| 27 | random*:ti,ab OR crossover*:ti,ab OR 'cross over*':ti,ab OR factorial*:ti,ab OR placebo*:ti,ab OR volunteer*:ti,ab | 1,507,810 |
| 28 | ((singl* OR doubl* OR trebl* OR tripl*) NEAR/5 (blind* OR mask*)):ab,ti | 202,353 |
| 29 | 'animal'/de | 1,780,507 |
| 30 | 'nonhuman'/de | 5,078,041 |
| 31 | 'human'/de | 18,126,323 |
| 32 | 29 OR 30 NOT (31 AND (29 OR 30)) | 5,266,209 |
| 33 | 'major clinical study'/exp | 2,778,248 |
| 34 | 'controlled study'/exp | 5,677,017 |
| 35 | 'clinical article'/exp | 1,689,490 |
| 36 | random* OR trial* OR control* OR study OR compar* OR placebo* OR blind* OR doubl* | 17,232,118 |
| 37 | 'cohort analysis'/exp | 286,850 |
| 38 | 19 OR 20 OR 21 OR 22 OR 23 OR 24 OR 25 OR 26 OR 27 OR 28 OR 33 OR 34 OR 35 OR 36 OR 37 | 17,641,671 |
| 39 | 38 NOT 32 | 14,123,054 |
| 40 | 18 AND 39 | 6,815 |
| 43 | tavi:ab OR 'ta tavi':ab OR tavr:ab OR pavi:ab OR pavr | 9,000 |
| 44 | transcutan* OR transarterial* OR percutan* OR transcatheter* OR transkatheter* OR transapical* OR transfemor* OR transsubclav* OR transaort* OR 'trans cutan*' OR 'trans arterial*' OR 'trans catheter*' OR 'trans katheter*' OR 'trans apical*' OR 'trans femor*' OR 'trans subclav*' OR 'trans aort*' AND aort* AND valve* AND (implant* OR insert* OR replace*) | 17,319 |
| 45 | 43 OR 44 | 17,571 |
| 46 | 'meta analysis' OR 'meta-analysis'/exp | 189,990 |
| 47 | 45 AND 46 | 407 |
| 48 | surg*:ti OR operat*:ti | 815,919 |
| 49 | ballon* OR valvuloplast* | 13,407 |
| 50 | 'medical treatment' | 57,280 |
| 51 | 'aorta stenosis'/exp/dm_dt,dm_th | 2,806 |
| 52 | 'aorta valve stenosis'/dm_dt,dm_th | 1,271 |
| 53 | 'standard therapy' | 17,105 |
| 54 | 'treatment outcome'/exp AND (compar* OR versus OR vs) | 485,890 |
| 55 | 48 OR 49 OR 50 OR 51 OR 52 OR 53 OR 54 | 1,339,982 |
| 56 | 47 AND 55 | 166 |
| 57 | 40 OR 56 | 6,875 |
| 62 | 57 AND [2002-2017]/py | 6,715 |
| 63 | 62 AND [27-1-2017]/sd NOT [6-6-2017]/sd | 410 |

**Cochrane Central Register of Controlled Trials (June 6, 2017)**

| # |  | Results |
| --- | --- | --- |
| 1 | exp Cardiac Catheterization/ | 2329 |
| 2 | exp Heart Valve Prosthesis/ | 473 |
| 3 | exp Heart Valve Prosthesis Implantation/ | 539 |
| 4 | 2 or 3 | 890 |
| 5 | exp Aortic Valve/ | 365 |
| 6 | exp Aortic Valve Stenosis/ | 510 |
| 7 | 5 or 6 | 751 |
| 8 | 1 and 4 and 7 | 72 |
| 9 | ((transcutan* or transarterial* or percutan* or transcatheter* or transkatheter* or transapical* or transfemor* or transsubclav* or transaort* or trans-cutan* or trans-arterial* or trans-catheter* or trans-katheter* or trans-apical* or trans-femor* or trans-subclav* or trans-aort*) and aort* and valve* and (implant* or insert* or replace*)).ti. | 359 |
| 10 | ((transcutan* or transarterial* or percutan* or transcatheter* or transkatheter* or transapical* or transfemor* or transsubclav* or transaort* or trans-cutan* or trans-arterial* or trans-catheter* or trans-katheter* or trans-apical* or trans-femor* or trans-subclav* or trans-aort*) and aort* and valve* and (implant* or insert* or replace*)).ab. | 461 |
| 11 | (tavi or ta-tavi or tavr or pavi or pavr).ti. | 67 |
| 12 | (tavi or ta-tavi or tavr or pavi or pavr).ab. | 421 |
| 13 | (aort* adj3 stenos*).ti,ab,sh,hw. | 849 |
| 14 | (9 or 10 or 11 or 12) and 13 | 354 |
| 15 | 8 or 14 | 366 |
| 16 | ((transcutan* or transarterial* or percutan* or transcatheter* or transkatheter* or transapical* or transfemor* or transsubclav* or transaort* or trans-cutan* or trans-arterial* or trans-catheter* or trans-katheter* or trans-apical* or trans-femor* or trans-subclav* or trans-aort*) and aort* and valve* and (implant* or insert* or replace*)).mp. | 522 |
| 17 | (tavi or ta-tavi or tavr or pavi or pavr).mp. | 427 |
| 18 | 16 or 17 | 542 |
| 19 | meta-analysis.mp. or exp Meta-Analysis/ | 5239 |
| 20 | 18 and 19 | 17 |
| 21 | (surg* or operat*).ti. | 46576 |
| 22 | (ballon* or valvuloplast*).mp. [mp=title, original title, abstract, mesh headings, heading words, keyword] | 228 |
| 23 | medical treatment.mp. | 2984 |
| 24 | exp Aortic Valve Stenosis/dt, th [Drug Therapy, Therapy] | 155 |
| 25 | standard therapy.mp. [mp=title, original title, abstract, mesh headings, heading words, keyword] | 2801 |
| 26 | exp Treatment Outcome/ and (compar* or versus or vs).mp. [mp=title, original title, abstract, mesh headings, heading words, keyword] | 77856 |
| 27 | 21 or 22 or 23 or 24 or 25 or 26 | 124685 |
| 28 | 20 and 27 | 7 |
| 29 | 15 or 28 | 367 |
| 30 | limit 29 to yr="2002 -Current" | 361 |
| 31 | limit 30 to yr="2016 -Current" | 147 |

**Excluded full-text articles**

Overall, we excluded 137 full-text articles due to the following reasons:

**Ineligible publication type: 1**

Shah A, Brambley H, Curtis M, Mullen M, Delahunty N, Yap J, Smith A, Montgomery H, Sanders J: **Postoperative morbidity after surgical aortic valve replacement or transcatheter valve implantation: a prospective cohort study**. *Intensive Care Med* 2015, **41**(9):1721-1722.

**Abstract only: 44**

[

Dumonteil N, Tchetche D, Monteil B, Bongard V, Marcheix B, Berthoumieu P, Massabuau P, Soula P, Fajadet J, Carrié D: **Non-randomised comparison of 30-day safety and effectiveness of surgical aortic valve replacement and transcatheter aortic valve implantation for high risk patients with severe aortic stenosis**. *Archives of Cardiovascular Diseases Supplements* 2011, **3**(1):54.

Dumonteil N, Tchetche D, Marcheix B, Bongard V, Berthoumieu P, Gautier M, Monteil B, Soula P, Fajadet J, Carrié D: **One year all-cause mortality after surgical aortic valve replacement and transcatheter aortic valve implantation for the treatment of severe aortic stenosis in high-risk patients: A two-centre study**. *Archives of Cardiovascular Diseases Supplements* 2012, **4**(1):60.

Seccareccia F, D'Errigo P, Tamburino C, Santini F, Barbanti M, Onorati F, Santoro G, Grossi C, Ranucci M, Covello RD: **Final results of the Italian OBSERVANT study: Comparative effectiveness evaluation of TAVI vs SAVR in a real world setting**. *European Heart Journal: Acute Cardiovascular Care* 2013, **2**:136.

Ohlmann P, Kindo M, Olivier M, Hoang T, Gros H, Cristinar M, Petit H, Hess S, Zerbib C, Kremer H *et al*: **Comparison of transcatheter aortic valve implantation (TAVI) and surgical aortic valve replacement in very high risk patients, monocentric registry of an early experience**. *Archives of Cardiovascular Diseases Supplements* 2014, **6**:68.

Boergermann J, Emmel E, Scholtz S, Ensminger S, Scholtz W, Becker T, Zittermann A, Horstkotte D, Kuss O, Gummert JF: **Conventional vs. Transapical vs. Transfemoral aortic valve replacement-Real world comparison of 3751 patients in propensity score matched groups**. *Eur Heart J* 2015, **36**:270.

Börgermann J, Emmel E, Scholtz S, Ensminger S, Scholtz W, Becker T, Zittermann A, Horstkotte D, Kuss O, Gummert JF: **Conventional vs. Transcatheter aortic valve replacement-propensity score adjusted comparison of 3,751 unselected patients**. *Circulation* 2015, **132**.

Castrodeza J, Amat Santos IJ, Blanco M, Lopez J, Di Stefano A, Tobar J, Cortes C, Revilla A, Gomez I, San Roman A: **Matched comparison of surgical aortic valve replacement versus transcatheter valve implantation in intermediate to low risk aortic stenosis patients**. *Eur Heart J* 2015, **36**:1050-1051.

Chakraborty A, Chatterjee S, Sardar P, Kumbhani DJ, Mukherjee D, Giri JS: **Transcatheter versus surgical aortic valve replacement in chronic obstructive pulmonary disease: An analysis of the national inpatient sample database**. *American Journal of Respiratory and Critical Care Medicine* 2015, **191**.

Chatterjee S, Sardar P, Mukherjee D, Kumbhani D, Giri J: **Mortality, expense and length of stay with transcatheter versus surgical aortic valve replacement in the first year after approval in the us: An analysis of the national inpatient sample database**. *J Am Coll Cardiol* 2015, **65**(10):A1706.

Chung J, Postoev A, Filatov A, Ladoris L, Farinas A, Cruz Pico CX, Postoev A, Ibikunle C, Sanni A: **Postoperative outcomes of surgical sutureless aortic valve replacement vs transcatheter aortic valve implantation for severe symptomatic aortic stenosis**. *J Am Coll Surg* 2015, **221**(4):S26.

Emmel E, Kuss O, Scholtz S, Ensminger S, Fujita B, Scholtz W, Becker T, Zittermann A, Horstkotte D, Gummert JF *et al*: **Comparison of conventional versus transcatheter aortic valve implantation-analysis of 3,751 patients in propensity score matched groups**. *EuroIntervention* 2015.

Folesani G, Savini C, Cefarelli M, Alfonsi J, Berretta P, Jafrancesco G, Di Eusanio M, Saia F, Reggiani MLB, Marzocchi A *et al*: **Treatment of aortic valve stenosis in high-risk patients: Role of the new surgical options**. *Innovations: Technology and Techniques in Cardiothoracic and Vascular Surgery* 2015, **10**:S74-S75.

Khan AR, Khan S, Simo H, Luni FK, Abdulhak AAB, Bavishi C, Riaz H, Flaherty MP: **Efficacy and safety of transcatheter aortic valve replacement in low-moderate surgical risk patients: A systematic review and meta-analysis**. *Circulation* 2015, **132**.

Koulova A, Chatterjee S, Sardar P, Giri J, DiNicolantonio J, Biondi-Zoccai G, Feldman D, Bangalore S: **Transcatheter versus Surgical aortic valve replacement in end stage renal disease: An analysis of the nationwide inpatient sample database**. *J Am Coll Cardiol* 2015, **65**(10):A1948.

Kresoja KP, Maier R, Stoschitzky G, Binder J, Kapl M, Marte W, Brussee H, Luha O, Schmidt A: **Percutaneous vs. surgical treatment strategy in patients with severe aortic stenosis and concomitant coronary artery disease: A 30-day outcome single-centre analysis**. *EuroIntervention* 2015.

Moat N, Duncan A, Stephens SV, Hickey GL, Cunningham D, De Belder M, Blackman DJ, Hildick-Smith D, Bridgewater B, Ludman P: **Aortic valve intervention in octogenarians in the “TAVI-era”: Analysis of the UK national adult cardiac surgery audit registry and the UK transcatheter aortic valve implantation (TAVI) registry between 2006 and 2012**. *J Am Coll Cardiol* 2015, **66**(15):B273.

Mohammadi S, Dumont E, Rodes-Cabau J, Voisine P, Doyle D, Charbonneau E, Dagenais F, Kalavrouziotis D: **Results of surgical aortic valve replacement in octogenarians turned down for trans-catheter aortic valve replacement by the multidisciplinary heart team**. *Can J Cardiol* 2015, **31**(10):S179.

Muneretto C, De Bonis M, Bisleri G, Alfieri O, Di Bartolomeo R, Rambaldini M, Maureira JP, Laborde F, Tespili M, Folliguet T: **Which is the optimal strategy for patients with severe aortic stenosis and intermediate-high risk profile? A multicenter propensity-score analysis in 991 consecutive patients**. *Eur Heart J* 2015, **36**:1050.

Ohno Y, Attizzani GF, Barbanti M, D'Errigo P, Grossi C, Covello RD, Francesco O, Francesco S, Ranucci M, Rosato S *et al*: **Transcatheter aortic valve implantation versus surgical aortic valve replacement in severe aortic stenosis patients undergoing chronic haemodialysis: Insights from Italian OBSERVANT study**. *EuroIntervention* 2015.

Pietzsch JB, Busca R, Geisler BP: **Adoption of transcatheter aortic valve replacement in Germany: Utilization patterns and case volumes compared to surgical aortic valve replacement in the period 2009-2013**. *Value in Health* 2015, **18**(7):A370.

Reinöhl J, Kaier K, Reinecke H, Schmoor C, Frankenstein L, Beyersdorf F, Bode C, Zehender M: **Surgical and transcatheter aortic valve replacement in Germany from 2007 to 2012: In-hospital outcomes of 96,149 patients**. *EuroIntervention* 2015.

Sakic A, Heinz A, Semsroth S, Kilo J, Bartel T, Friedrich G, Müller S, Franz WM, Müller L, Grimm M *et al*: **Transcatheter aortic valve implantation versus surgical aortic valve replacement in high-risk patients older than 75 years: A propensity-matched analysis in a non-diagnosis-related-group- based reimbursement system**. *Innovations: Technology and Techniques in Cardiothoracic and Vascular Surgery* 2015, **10**:S80-S81.

Spinetto PV, Briceno D, Mignatti A, Al-Bawardy R, Villablanca S, Slovut D: **Transcatheter aortic valve replacement versus surgical aortic valve replacement for severe aortic stenosis: Meta-analysis of randomized controlled trials**. *J Am Coll Cardiol* 2015, **65**(10):A1878.

Thakkar B, Patel A, Bhimani R, Patel NJ, Bhatt P, Savani C, Patel A, Patel S, Sonani R, Solanki S *et al*: **Transcatheter aortic valve replacement versus surgical aortic valve replacement in patients with cirrhosis: A propensity score matched analysis**. *Circulation* 2015, **132**.

Wilson V, Wong T, Tamuno P, Pasupati S, Nair R: **Safety and efficacy of transcatheter aortic valve implantation compared with surgical aortic valve replacement in older high-risk patients with severe aortic stenosis - A single centre study**. *Heart Lung and Circulation* 2015, **24**:S300-S301.

Agrawal Y, Jacob C, Konda MK, Panaich S, Kalavakunta J, Gupta V: **Review of transcatheter aortic valve replacement between the years 2012 and 2013: Nationwide inpatient sample data analysis**. *J Am Coll Cardiol* 2016, **67**(13):341.

Auensen A, Hussain AI, Bye J, Hansen M, Falk RS, Pettersen KI, Gullestad L: **Comparing one-year outcomes beyond mortality in operated versus non-operated patients with severe aortic valve stenosis**. *Eur J Heart Fail* 2016, **18**:223.

Baron S, Alu M, Thourani V, Kodali S, Arnold S, Chinnakondepali K, Magnuson E, Wang K, Pichard A, Babaliaros V *et al*: **Effect of sapien-3 transcatheter valve implant on health status in patients with severe aortic stenosis at intermediate surgical risk: Results from the PARTNER S3i trial**. *J Am Coll Cardiol* 2016, **68**(18):B14.

Boissonnet CP, Giorgi M, Thierer J, Guetta JN, Giglio N, Micone P, Gonzalez CD: **Transcatheter aortic valve replacement in south-America: A meta-analysis of real-life outcomes**. *Value in Health* 2016, **19**(3):A298-A299.

Büttner S, Weiler H, Zöller C, Patyna S, Honold J, Papadopoulos N, Geiger H, Hauser IA, Vasa-Nicotera M, Fichtlscherer S: **Comparison of surgical versus interventional treatment of aortic valve stenosis in kidney transplant recipients**. *J Am Coll Cardiol* 2016, **68**(18):B274.

Garg A, Sharma A, Agrawal S, Virmani D, Kostis J, Singal D: **Transcatheter aortic valve replacement versus surgical valve replacement for low to intermediate surgical-risk patients: A metaanalysis of 5,346 patients**. *J Am Coll Cardiol* 2016, **68**(18):B295-B296.

Grønlykke L, Ihlemann N, Nilsson JC, Kjaergaard J, Korshin A, Gustafsson F, Thyregod HG, Søndergaard L, Ravn HB: **Echocardiographic changes in right ventricular function after transcatheter versus surgical aortic valve replacement for severe aortic valve stenosis**. *Journal of Cardiothoracic and Vascular Anesthesia* 2016, **30**:S31-S32.

Khounlaboud M, Donal E, Auffret V, Ingels A, Flecher E, Verhoye JP, Daubert JC, Le Breton H, Mabo P, Leguerrier A: **Comparison of pre-and post-operative characteristics in octogenarians having isolated surgical aortic valve replacement before versus after introduction of transcatheter aortic valve implantation**. *Archives of Cardiovascular Diseases Supplements* 2016, **Conference: 26es Journees Europeennes de la Societe Francaise de Cardiologie Paris France. Conference Start:. 20160113 Conference End: 20160116. Conference Publication:**(var.pagings) 8 (1 SUPPL. 1):54-55.

Marta Zaleska-Kociecka M, Skrobisz A, Wojtkowska I, Grabowski M, Dabrowski M, Kusmierski K, Imiela J, Stepinska J: **Is there any difference in interplay between kidney and heart after TAVR or SAVR?** *Eur J Heart Fail* 2016, **18**:372-373.

Mattos AZ, Schroder DA, Colpani V, Restelatto LM, Ribeiro RA: **Transcatheter aortic valve implantation for inoperable severe aortic stenosis-a meta-analysis**. *Value in Health* 2016, **19**(7):A686.

Muneretto C, Alfieri O, Bisleri G, De Bonis M, Di Bartolomeo R, Savini C, Folesani G, Di Bacco L, Maureira JP, Laborde F *et al*: **Treatment options in intermediate-high risk patients with aortic stenosis and renal disfunction: The European multicenter propensity match study comparing tavr versus conventional avr surgery**. *Eur Heart J* 2016, **37**:147.

Noutsias M, Ak A, Porokhovnikov I, Kuethe F, Schlattmann P: **Comparison of transcatheter aortic valve implantation versus surgical aortic valve replacement and medical treatment: A systematic review and meta-analysis**. *Eur Heart J* 2016, **37**:1399.

Panoulas V, Thyregod HG, Nihoyannopoulos P, Sen S, Ariff B, Gopalan D, Sutaria N, Bicknell C, Malik I, Francis D *et al*: **Increased survival of females with severe aortic stenosis after transcatheter aortic valve implantation compared to surgical aortic valve replacement; a meta-analysis of randomised controlled studies**. *Eur Heart J* 2016, **37**:72.

Salizzoni S, D'Errigo P, Barbero C, Ferrigno L, Rosato S, Barbanti M, Tamburino C, Rinaldi M, Seccareccia F: **Futility in transcatheter aortic valve implantation: Result from the Italian multicenter OBSERVANT Study**. *J Am Coll Cardiol* 2016, **68**(18):B273.

Sardar P, Chatterjee S, Kundu A, Nairooz R, Owan T, Tandar A, Welt FG: **Transcatheter versus surgical aortic valve replacement in diabetic patients: An analysis of the national inpatient sample database**. *Catheterization and Cardiovascular Interventions* 2016, **87**:S153-S154.

Sardar P, Kundu A, Chatterjee S, Feldman D, Owan T, Nairooz R, Feldman T, Abbott JD, Elmariah S: **TCT-756 Transcatheter versus Surgical Aortic-Valve Replacement in Intermediate-Risk Patients: Evidence from a Meta-analysis**. *J Am Coll Cardiol* 2016, **68**(18S):B305.

Schymik G, Varsami C, Tzamalis P, Herzberger V, Bergmann J, Bramlage P, Schröfel H, Würth A, Conzelmann L, Gonska BD *et al*: **TAVIK registry: Mortality and morbidity of low-risk patients. A comparison of TAVI with conventional surgery: Results of a single-centre experience**. *EuroIntervention* 2016:413.

Turagam M, Velagapudi P, Agrawal H, Mittal M, Katta N, Abbott JD, Aggarwal K: **Pacemaker implantation in transcatheter aortic valve replacement vs. Sutureless surgical aortic valve replacement: A meta-analysis**. *J Am Coll Cardiol* 2016, **68**(18):B101.

Yammine MB, Ejiofor J, McGurk S, Norman A, Shekar P, Cohn L, Aranki S, Shah P, Kaneko T: **Benefits of transcatheter aortic valve replacement in patients with unclampable calcified aorta: Quantifying reduced mortality risk in the TAVR era**. *J Am Coll Cardiol* 2016, **67**(13):2223.

**Ineligible population: 1**

Genereux P, Stone G, O'Gara P, Gravel GM, Redfors B, Giustino G, Pibarot P, Bax J, Bonow R, Leon M: **Early aortic valve replacement versus a conservative strategy for asymptomatic severe aortic stenosis: Meta-analysis of observational studies**. *J Am Coll Cardiol* 2016, **67**(13):2210.

**Ineligible intervention: 0**

**Ineligible or no control group: 1**

Barbanti M, Schiltgen M, Verdoliva S, Bosmans J, Bleiziffer S, Gerckens U, Wenaweser P, Brecker S, Gulino S, Tamburino C *et al*: **Three-Year Outcomes of Transcatheter Aortic Valve Implantation in Patients With Varying Levels of Surgical Risk (from the CoreValve ADVANCE Study)**. *Am J Cardiol* 2016, **117**(5):820-827.

**Ineligible outcome or no appropriate outcome data: 7**

Bagur R, Webb JG, Nietlispach F, Dumont E, De Larochelliere R, Doyle D, Masson JB, Gutierrez MJ, Clavel MA, Bertrand OF *et al*: **Acute kidney injury following transcatheter aortic valve implantation: predictive factors, prognostic value, and comparison with surgical aortic valve replacement**. *Eur Heart J* 2010, **31**(7):865-874.

Bagur R, Rodes-Cabau J, Gurvitch R, Dumont E, Velianou JL, Manazzoni J, Toggweiler S, Cheung A, Ye J, Natarajan MK *et al*: **Need for permanent pacemaker as a complication of transcatheter aortic valve implantation and surgical aortic valve replacement in elderly patients with severe aortic stenosis and similar baseline electrocardiographic findings**. *JACC Cardiovasc Interv* 2012, **5**(5):540-551.

Alassar A, Roy D, Valencia O, Brecker S, Jahangiri M: **Cerebral embolization during transcatheter aortic valve implantation compared with surgical aortic valve replacement**. *Interactive Cardiovascular and Thoracic Surgery* 2013, **17**:S134-S135.

Uddin A, Fairbairn TA, Djoukhader IK, Igra M, Kidambi A, Motwani M, Herzog B, Ripley DP, Musa TA, Goddard AJ *et al*: **Consequence of cerebral embolism after transcatheter aortic valve implantation compared with contemporary surgical aortic valve replacement: effect on health-related quality of life**. *Circ* 2015, **8**(3):e001913.

Amofah HA, Brostrom A, Fridlund B, Bjorvatn B, Haaverstad R, Hufthammer KO, Kuiper KK, Ranhoff AH, Norekval TM, Investigators C: **Sleep in octogenarians during the postoperative phase after transcatheter or surgical aortic valve replacement**. *European Journal of Cardiovascular Nursing* 2016, **15**(2):168-177.

Piazza N, van Gameren M, Juni P, Wenaweser P, Carrel T, Onuma Y, Gahl B, Hellige G, Otten A, Kappetein AP *et al*: **A comparison of patient characteristics and 30-day mortality outcomes after transcatheter aortic valve implantation and surgical aortic valve replacement for the treatment of aortic stenosis: a two-centre study**. *EuroIntervention* 2009, **5**(5):580-588.

Tamburino C, Barbanti M, Capodanno D, Mignosa C, Gentile M, Aruta P, Pistritto AM, Bonanno C, Bonura S, Cadoni A *et al*: **Comparison of complications and outcomes to one year of transcatheter aortic valve implantation versus surgical aortic valve replacement in patients with severe aortic stenosis**. *Am J Cardiol* 2012, **109**(10):1487-1493.

**Ineligible study design: 49**

Ewe SH, Ajmone Marsan N, Pepi M, Delgado V, Tamborini G, Muratori M, Ng AC, van der Kley F, de Weger A, Schalij MJ *et al*: **Impact of left ventricular systolic function on clinical and echocardiographic outcomes following transcatheter aortic valve implantation for severe aortic stenosis**. *Am Heart J* 2010, **160**(6):1113-1120.

Grant SW, Devbhandari MP, Grayson AD, Dimarakis I, Kadir I, Saravanan DM, Levy RD, Ray SG, Bridgewater B: **What is the impact of providing a transcatheter aortic valve implantation service on conventional aortic valve surgical activity: patient risk factors and outcomes in the first 2 years**. *Heart* 2010, **96**(20):1633-1637.

Ranucci M, Guarracino F, Castelvecchio S, Baldassarri R, Covello RD, Landoni G, Group ASR: **Surgical and transcatheter aortic valve procedures. The limits of risk scores**. *Interact Cardiovasc Thorac Surg* 2010, **11**(2):138-141.

Ben-Dor I, Gaglia MA, Maluenda G, Mahmoudi M, Sardi G, Wakabayashi K, Gonzalez MA, Romaguera R, Laynez-Carnicero A, Torguson R *et al*: **Outcome of patients with severe symptomatic aortic stenosis screened for transcatheter aortic valve implantation and treated with surgical aortic valve replacement**. *J Am Coll Cardiol* 2011, **1)**:E1372.

Ben-Dor I, Goldstein SA, Pichard AD, Satler LF, Maluenda G, Li Y, Syed AI, Gonzalez MA, Gaglia MA, Jr., Wakabayashi K *et al*: **Clinical profile, prognostic implication, and response to treatment of pulmonary hypertension in patients with severe aortic stenosis**. *Am J Cardiol* 2011, **107**(7):1046-1051.

Pilgrim T, Wenaweser P, Meuli F, Huber C, Stortecky S, Seiler C, Zbinden S, Meier B, Carrel T, Windecker S: **Clinical outcome of high-risk patients with severe aortic stenosis and reduced left ventricular ejection fraction undergoing medical treatment or TAVI**. *PLoS ONE* 2011, **6**(11):e27556.

Saia F, Marrozzini C, Moretti C, Ciuca C, Taglieri N, Bordoni B, Dall'Ara G, Alessi L, Lanzillotti V, Bacchi-Reggiani ML *et al*: **The role of percutaneous balloon aortic valvuloplasty as a bridge for transcatheter aortic valve implantation**. *EuroIntervention* 2011, **7**(6):723-729.

Stohr R, Dohmen G, Herpertz R, Brehmer K, Aktug O, Koos R, Altiok E, Stegemann E, Autschbach R, Marx N *et al*: **Thirty-day outcome after transcatheter aortic valve implantation compared with surgical valve replacement in patients with high-risk aortic stenosis: a matched comparison**. *Coron Artery Dis* 2011, **22**(8):595-600.

Tissot CM, Attias D, Himbert D, Ducrocq G, Iung B, Dilly MP, Juliard JM, Lepage L, Detaint D, Messika-Zeitoun D *et al*: **Reappraisal of percutaneous aortic balloon valvuloplasty as a preliminary treatment strategy in the transcatheter aortic valve implantation era**. *EuroIntervention* 2011, **7**(1):49-56.

Wenaweser P, Pilgrim T, Kadner A, Huber C, Stortecky S, Buellesfeld L, Khattab AA, Meuli F, Roth N, Eberle B *et al*: **Clinical outcomes of patients with severe aortic stenosis at increased surgical risk according to treatment modality**. *J Am Coll Cardiol* 2011, **58**(21):2151-2162.

Heuvelman HJ, van Geldorp MW, Kappetein AP, Geleijnse ML, Galema TW, Bogers AJ, Takkenberg JJ: **Clinical course of patients diagnosed with severe aortic stenosis in the Rotterdam area: insights from the AVARIJN study**. *Neth Heart J* 2012, **20**(12):487-493.

Kempny A, Diller GP, Kaleschke G, Orwat S, Funke A, Schmidt R, Kerckhoff G, Ghezelbash F, Rukosujew A, Reinecke H *et al*: **Impact of transcatheter aortic valve implantation or surgical aortic valve replacement on right ventricular function**. *Heart* 2012, **98**(17):1299-1304.

Nuis RJ, Dager AE, van der Boon RM, Jaimes MC, Caicedo B, Fonseca J, Van Mieghem NM, Benitez LM, Umana JP, O'Neill WW *et al*: **Patients with aortic stenosis referred for TAVI: treatment decision, in-hospital outcome and determinants of survival**. *Neth Heart J* 2012, **20**(1):16-23.

D'Onofrio A, Alfieri OR, Cioni M, Alamanni F, Fusari M, Tarzia V, Rizzoli G, Gerosa G: **The impact of transcatheter aortic valve implantation on patients' profiles and outcomes of aortic valve surgery programmes: a multi-institutional appraisal**. *Interact Cardiovasc Thorac Surg* 2013, **16**(5):608-611.

Dvir D, Sagie A, Porat E, Assali A, Shapira Y, Vaknin-Assa H, Shafir G, Bental T, Nevzorov R, Battler A *et al*: **Clinical profile and outcome of patients with severe aortic stenosis at high surgical risk: single-center prospective evaluation according to treatment assignment**. *Catheter Cardiovasc Interv* 2013, **81**(5):871-881.

Quick S, Speiser U, Pfluecke C, Youssef A, Sveric K, Strasser R, Ibrahim K: **Aortic stenosis: right and left ventricular function in the early postprocedural phase. Comparison between transcatheter and surgical aortic valve implantation**. *Acta Cardiol* 2013, **68**(6):583-589.

Martinez-Selles M, Gomez Doblas JJ, Carro Hevia A, Garcia de la Villa B, Ferreira-Gonzalez I, Alonso Tello A, Andion Ogando R, Ripoll Vera T, Arribas Jimenez A, Carrillo P *et al*: **Prospective registry of symptomatic severe aortic stenosis in octogenarians: a need for intervention**. *J Intern Med* 2014, **275**(6):608-620.

Nagaraja V, Raval J, Eslick GD, Ong AT: **Transcatheter versus surgical aortic valve replacement: a systematic review and meta-analysis of randomised and non-randomised trials**. *Open Heart* 2014, **1**(1):e000013.

Nguyen TC, Babaliaros VC, Razavi SA, Kilgo PD, Devireddy CM, Leshnower BG, Mavromatis K, Guyton RA, Kanitkar M, Lerakis S *et al*: **Transcatheter aortic valve replacement has improved short-term but similar midterm outcomes in isolated aortic valve replacement after prior coronary artery bypass grafting**. *Ann Thorac Surg* 2014, **98**(4):1316-1324.

Tanawuttiwat T, O'Neill BP, Cohen MG, Chinthakanan O, Heldman AW, Martinez CA, Alfonso CE, Mitrani RD, Macon CJ, Carrillo RG *et al*: **New-onset atrial fibrillation after aortic valve replacement: comparison of transfemoral, transapical, transaortic, and surgical approaches**. *J Am Coll Cardiol* 2014, **63**(15):1510-1519.

Cardoso RN, Ansari MM, Mendirichaga R, Garcia DC, Brinster D, Patel NC, Scheinerman SJ, Martucci G, Piazza N: **Heart failure functional classification following transcatheter versus surgical aortic valve replacement: A meta-analysis of three randomized controlled trials**. *J Am Coll Cardiol* 2015, **66**(15):B299.

Khounlaboud M, Donal E, Auffret V, Anselmi A, Ingels A, Flecher E, Verhoye JP, Daubert C, Le Breton H, Mabo P *et al*: **Comparison of Preoperative and Postoperative Characteristics in Octogenarians Having Isolated Surgical Aortic Valve Replacement Before Versus After Introduction of Transcatheter Aortic Valve Implantation**. *Am J Cardiol* 2015, **116**(6):933-937.

O'Sullivan CJ, Englberger L, Hosek N, Heg D, Cao D, Stefanini GG, Stortecky S, Gloekler S, Spitzer E, Tuller D *et al*: **Clinical outcomes and revascularization strategies in patients with low-flow, low-gradient severe aortic valve stenosis according to the assigned treatment modality**. *JACC Cardiovasc Interv* 2015, **8**(5):704-717.

Phan K, Wong S, Phan S, Ha H, Qian P, Yan TD: **Transcatheter Aortic Valve Implantation (TAVI) in Patients With Bicuspid Aortic Valve Stenosis--Systematic Review and Meta-Analysis**. *Heart Lung Circ* 2015, **24**(7):649-659.

Pilgrim T, Englberger L, Rothenbuhler M, Stortecky S, Ceylan O, O'Sullivan CJ, Huber C, Praz F, Buellesfeld L, Langhammer B *et al*: **Long-term outcome of elderly patients with severe aortic stenosis as a function of treatment modality**. *Heart* 2015, **101**(1):30-36.

Ribera A, Slof J, Andrea R, Falces C, Gutierrez E, Del Valle-Fernandez R, Moris-de la Tassa C, Mota P, Oteo JF, Cascant P *et al*: **Transfemoral transcatheter aortic valve replacement compared with surgical replacement in patients with severe aortic stenosis and comparable risk: cost-utility and its determinants**. *Int J Cardiol* 2015, **182**:321-328.

Wendt D, Al-Rashid F, Kahlert P, El-Chilali K, Demircioglu E, Neuhauser M, Liakopoulos O, Sebastian Dohle D, Erbel R, Jakob H *et al*: **Conventional aortic valve replacement or transcatheter aortic valve implantation in patients with previous cardiac surgery**. *J Cardiol* 2015, **66**(4):292-297.

Wong KYK, Malik T, Allgar V: **Transcatheter aortic valve implant vs surgical aortic valve replacement in low-to intermediate risk patients: A Meta-analysis**. *Eur Heart J* 2015, **36**:954-955.

Xiong TY, Liao YB, Zhao ZG, Xu YN, Wei X, Zuo ZL, Li YJ, Cao JY, Tang H, Jilaihawi H *et al*: **Causes of Death Following Transcatheter Aortic Valve Replacement: A Systematic Review and Meta-Analysis**. *J Am Heart Assoc* 2015, **4**(9):e002096.

Ad N, Holmes SD, Shuman DJ, Rongione AJ, Massimiano PS, Speir AM, Pritchard G, Yazdani S, Raybuck BD: **The Effect of Initiation of a Transcatheter Aortic Valve Replacement Program in the Treatment of Severe Aortic Stenosis**. *Seminars in Thoracic and Cardiovascular Surgery* 2016, **28**(2):353-360.

Ak A, Porokhovnikov I, Kuethe F, Noutsias M, Schlattmann P: **Comparison of transcatheter aortic valve implantation versus surgical aortic valve replacement and medical treatment: A systematic review and meta-analysis**. *Eur J Heart Fail* 2016, **18**:82.

Amato L, Parmelli E, Vecchi S, Minozzi S, Mitrova Z, De Palma R, Berti E, Davoli M: **[Transcatheter aortic valve implantation: a systematic review of the literature on efficacy and safety data]**. *Recenti Prog Med* 2016, **107**(1):25-38.

Boissonnet CP, Giorgi M, Thierer J, Guetta JN, Giglio N, Micone P, Gonzalez CD: **Transcatheter aortic valve replacement in south-America: A meta-analysis of real-life outcomes**. *Value in Health* 2016, **19**(3):A298-A299.

Gaede L, Kim WK, Blumenstein J, Liebetrau C, Dörr O, Nef H, Hamm C, Walther T, Achenbach S, Elsässer A *et al*: **Temporal trends in transcatheter and surgical aortic valve replacement: An analysis of aortic valve replacements in Germany during 2012–2014**. *Herz* 2016:1-8.

González-Saldivar H, Rodriguez-Pascual C, De La Morena G, Fernández-Golfín C, Amorós C, Alonso MB, Dolz LM, Solé AA, Guzmán-Martínez G, Gómez-Doblas JJ *et al*: **Comparison of 1-Year Outcome in Patients with Severe Aorta Stenosis Treated Conservatively or by Aortic Valve Replacement or by Percutaneous Transcatheter Aortic Valve Implantation (Data from a Multicenter Spanish Registry)**. *Am J Cardiol* 2016, **118**(2):244-250.

Khan AR, Khan S, Riaz H, Luni FK, Simo H, Bin Abdulhak A, Bavishi C, Flaherty M: **Efficacy and safety of transcatheter aortic valve replacement in intermediate surgical risk patients: A systematic review and meta-analysis**. *Catheterization and Cardiovascular Interventions* 2016, **88**(6):934-944.

MacDonald ZD, Yousef A, Simard T, Russo JJ, Feder J, Froeschl MV, Dick A, Glover C, Burwash IG, Hibbert B *et al*: **Transcatheter aortic valve implantation (TAVI) in native aortic valve regurgitation: A systematic review**. In*.*, vol. 32; 2016: S100.

Mittal MK, Omran J, Firwana B, Velagapudi P, Gupta V, Kumar A, Aggarwal K: **Neurological outcomes and mortality associated with transcatheter versus surgical aortic valve replacement: A meta-analysis of studies**. *Catheterization and Cardiovascular Interventions* 2016, **87**:S149-S150.

Mollmann H, Bestehorn K, Bestehorn M, Papoutsis K, Fleck E, Ertl G, Kuck KH, Hamm C: **In-hospital outcome of transcatheter vs. surgical aortic valve replacement in patients with aortic valve stenosis: complete dataset of patients treated in 2013 in Germany**. *Clin* 2016, **105**(6):553-559.

Musa TA, Uddin A, Fairbairn TA, Dobson LE, Steadman CD, Kidambi A, Ripley DP, Swoboda PP, McDiarmid AK, Erhayiem B *et al*: **Right ventricular function following surgical aortic valve replacement and transcatheter aortic valve implantation: A cardiovascular MR study**. *Int J Cardiol* 2016, **223**:639-644.

Patel HJ, Likosky DS, Pruitt AL, Murphy ET, Theurer PF, Prager RL: **Aortic Valve Replacement in the Moderately Elevated Risk Patient: A Population-Based Analysis of Outcomes**. *Ann Thorac Surg* 2016, **102**(5):1466-1472.

Siemieniuk RA, Agoritsas T, Manja V, Devji T, Chang Y, Bala MM, Thabane L, Guyatt GH: **Transcatheter versus surgical aortic valve replacement in patients with severe aortic stenosis at low and intermediate risk: Systematic review and meta-analysis**. *BMJ (Online)* 2016, **354**.

Siontis GC, Praz F, Pilgrim T, Mavridis D, Verma S, Salanti G, Sondergaard L, Juni P, Windecker S: **Transcatheter aortic valve implantation vs. surgical aortic valve replacement for treatment of severe aortic stenosis: a meta-analysis of randomized trials**. *Eur Heart J* 2016, **37**(47):3503-3512.

Takagi H, Umemoto T, Group A: **Worse survival after transcatheter aortic valve implantation than surgical aortic valve replacement: A meta-analysis of observational studies with a propensity-score analysis**. *Int J Cardiol* 2016, **220**:320-327.

Takagi H, Umemoto T, Group A: **Sutureless aortic valve replacement may improve early mortality compared with transcatheter aortic valve implantation: A meta-analysis of comparative studies**. *J Cardiol* 2016, **67**(6):504-512.

Villablanca PA, Mathew V, Thourani VH, Rodes-Cabau J, Bangalore S, Makkiya M, Vlismas P, Briceno DF, Slovut DP, Taub CC *et al*: **A meta-analysis and meta-regression of long-term outcomes of transcatheter versus surgical aortic valve replacement for severe aortic stenosis**. *Int J Cardiol* 2016, **225**:234-243.

Wang N, Tsai YC, Niles N, Tchantchaleishvili V, Di Eusanio M, Yan TD, Phan K: **Transcatheter aortic valve implantation (TAVI) versus sutureless aortic valve replacement (SUAVR) for aortic stenosis: a systematic review and meta-analysis of matched studies**. *Journal of Thoracic Disease* 2016, **8**(11):3283-3293.

Wijeysundera HC, Li L, Braga V, Pazhaniappan N, Pardhan AM, Lian D, Leeksma A, Peterson B, Cohen EA, Forsey A *et al*: **Drivers of healthcare costs associated with the episode of care for surgical aortic valve replacement versus transcatheter aortic valve implantation**. *Open Heart* 2016, **3**(2):e000468.

Takagi H, Ando T, Umemoto T, Group A: **Direct and adjusted indirect comparisons of perioperative mortality after sutureless or rapid-deployment aortic valve replacement versus transcatheter aortic valve implantation**. *Int J Cardiol* 2017, **228**:327-334.

**Subgroup or analysis of previous published study: 17**

Reynolds MR, Magnuson EA, Lei Y, Leon MB, Smith CR, Svensson LG, Webb JG, Babaliaros VC, Bowers BS, Fearon WF *et al*: **Health-related quality of life after transcatheter aortic valve replacement in inoperable patients with severe aortic stenosis**. *Circulation* 2011, **124**(18):1964-1972.

Miller DC, Blackstone EH, Mack MJ, Svensson LG, Kodali SK, Kapadia S, Rajeswaran J, Anderson WN, Moses JW, Tuzcu EM *et al*: **Transcatheter (TAVR) versus surgical (AVR) aortic valve replacement: occurrence, hazard, risk factors, and consequences of neurologic events in the PARTNER trial**. *J Thorac Cardiovasc Surg* 2012, **143**(4):832-843.e813.

Reynolds MR, Magnuson EA, Lei Y, Wang K, Vilain K, Li H, Walczak J, Pinto DS, Thourani VH, Svensson LG *et al*: **Cost-effectiveness of transcatheter aortic valve replacement compared with surgical aortic valve replacement in high-risk patients with severe aortic stenosis: results of the PARTNER (Placement of Aortic Transcatheter Valves) trial (Cohort A)**. *J Am Coll Cardiol* 2012, **60**(25):2683-2692.

Reynolds MR, Magnuson EA, Wang K, Lei Y, Vilain K, Walczak J, Kodali SK, Lasala JM, O'Neill WW, Davidson CJ *et al*: **Cost-effectiveness of transcatheter aortic valve replacement compared with standard care among inoperable patients with severe aortic stenosis: results from the placement of aortic transcatheter valves (PARTNER) trial (Cohort B)**. *Circulation* 2012, **125**(9):1102-1109.

Reynolds MR, Magnuson EA, Wang K, Thourani VH, Williams M, Zajarias A, Rihal CS, Brown DL, Smith CR, Leon MB *et al*: **Health-related quality of life after transcatheter or surgical aortic valve replacement in high-risk patients with severe aortic stenosis: results from the PARTNER (Placement of AoRTic TraNscathetER Valve) Trial (Cohort A)**. *J Am Coll Cardiol* 2012, **60**(6):548-558.

Watt M, Mealing S, Eaton J, Piazza N, Moat N, Brasseur P, Palmer S, Busca R, Sculpher M: **Cost-effectiveness of transcatheter aortic valve replacement in patients ineligible for conventional aortic valve replacement**. *Heart* 2012, **98**(5):370-376.

Barbanti M, Webb JG, Hahn RT, Feldman T, Boone RH, Smith CR, Kodali S, Zajarias A, Thompson CR, Green P *et al*: **Impact of preoperative moderate/severe mitral regurgitation on 2-year outcome after transcatheter and surgical aortic valve replacement: insight from the Placement of Aortic Transcatheter Valve (PARTNER) Trial Cohort A**. *Circulation* 2013, **128**(25):2776-2784.

Dewey TM, Bowers B, Thourani VH, Babaliaros V, Smith CR, Leon MB, Svensson LG, Tuzcu EM, Miller DC, Teirstein PS *et al*: **Transapical aortic valve replacement for severe aortic stenosis: results from the nonrandomized continued access cohort of the PARTNER trial**. *Ann Thorac Surg* 2013, **96**(6):2083-2089.

Elmariah S, Palacios IF, McAndrew T, Hueter I, Inglessis I, Baker JN, Kodali S, Leon MB, Svensson L, Pibarot P *et al*: **Outcomes of transcatheter and surgical aortic valve replacement in high-risk patients with aortic stenosis and left ventricular dysfunction: results from the Placement of Aortic Transcatheter Valves (PARTNER) trial (cohort A)**. *Circ* 2013, **6**(6):604-614.

Hahn RT, Pibarot P, Stewart WJ, Weissman NJ, Gopalakrishnan D, Keane MG, Anwaruddin S, Wang Z, Bilsker M, Lindman BR *et al*: **Comparison of transcatheter and surgical aortic valve replacement in severe aortic stenosis: a longitudinal study of echocardiography parameters in cohort A of the PARTNER trial (placement of aortic transcatheter valves)**. *J Am Coll Cardiol* 2013, **61**(25):2514-2521.

Onorati F, D'Errigo P, Barbanti M, Rosato S, Covello DR, Maraschini A, Ranucci M, Grossi C, Santoro G, Tamburino C *et al*: **Results differ between transaortic and open surgical aortic valve replacement in women**. *Ann Thorac Surg* 2013, **96**(4):1336-1342.

Arnold SV, Reynolds MR, Lei Y, Magnuson EA, Kirtane AJ, Kodali SK, Zajarias A, Thourani VH, Green P, Rodes-Cabau J *et al*: **Predictors of poor outcomes after transcatheter aortic valve replacement: results from the PARTNER (Placement of Aortic Transcatheter Valve) trial**. *Circulation* 2014, **129**(25):2682-2690.

Pibarot P, Weissman NJ, Stewart WJ, Hahn RT, Lindman BR, McAndrew T, Kodali SK, Mack MJ, Thourani VH, Miller DC *et al*: **Incidence and sequelae of prosthesis-patient mismatch in transcatheter versus surgical valve replacement in high-risk patients with severe aortic stenosis: a PARTNER trial cohort--a analysis**. *J Am Coll Cardiol* 2014, **64**(13):1323-1334.

Passeri JJ, Elmariah S, Xu K, Inglessis I, Baker JN, Alu M, Kodali S, Leon MB, Svensson LG, Pibarot P *et al*: **Transcatheter aortic valve replacement and standard therapy in inoperable patients with aortic stenosis and low EF**. *Heart* 2015, **101**(6):463-471.

Skelding KA, Casale A, Yakubov S, Reardon M, Adams D, Popma J: **Transcatheter aortic valve replacement versus surgery in women at high risk for surgical aortic valve replacement: A subgroup analysis of the corevalve US high risk pivotal trial**. *J Am Coll Cardiol* 2015, **65**(10):A1946.

Lindman B, Abramowitz Y, Pibarot P, Quader N, Maniar H, Zajarias A, Arnold S, Hahn R, Elmariah S, Suri R *et al*: **Diabetes and clinical outcomes in intermediate risk patients randomized to transcatheter versus surgical aortic valve replacement: An analysis of the PARTNER 2A trial**. *J Am Coll Cardiol* 2016, **68**(18):B275.

Reardon MJ, Kleiman NS, Adams DH, Yakubov SJ, Coselli JS, Deeb GM, O'Hair D, Gleason TG, Lee JS, Hermiller JB, Jr. *et al*: **Outcomes in the Randomized CoreValve US Pivotal High Risk Trial in Patients With a Society of Thoracic Surgeons Risk Score of 7% or Less**. *JAMA Cardiol* 2016, **1**(8):945-949.

**Same study population or major overlap: 6**

D'Onofrio A, Messina A, Lorusso R, Alfieri OR, Fusari M, Rubino P, Rinaldi M, Di Bartolomeo R, Glauber M, Troise G *et al*: **Sutureless aortic valve replacement as an alternative treatment for patients belonging to the "gray zone" between transcatheter aortic valve implantation and conventional surgery: a propensity-matched, multicenter analysis**. *J Thorac Cardiovasc Surg* 2012, **144**(5):1010-1016.

Jilaihawi H, Doctor N, Chakravarty T, Kashif M, Mirocha J, Cheng W, Lill M, Nakamura M, Gheorghiu M, Makkar RR: **Major thrombocytopenia after balloon-expandable transcatheter aortic valve replacement: prognostic implications and comparison to surgical aortic valve replacement**. *Catheter Cardiovasc Interv* 2015, **85**(1):130-137.

D'Errigo P, Moretti C, D'Ascenzo F, Rosato S, Biancari F, Barbanti M, Santini F, Ranucci M, Miceli A, Tamburino C *et al*: **Transcatheter Aortic Valve Implantation Versus Surgical Aortic Valve Replacement for Severe Aortic Stenosis in Patients With Chronic Kidney Disease Stages 3b to 5**. *Ann Thorac Surg* 2016, **102**(2):540-547.

D'Errigo P, Ranucci M, Covello RD, Biancari F, Rosato S, Barbanti M, Onorati F, Tamburino C, Santoro G, Grossi C *et al*: **Outcome After General Anesthesia Versus Monitored Anesthesia Care in Transfemoral Transcatheter Aortic Valve Replacement**. *J Cardiothorac Vasc Anesth* 2016, **30**(5):1238-1243.

Fraccaro C, Tarantini G, Rosato S, Tellaroli P, D'Errigo P, Tamburino C, Onorati F, Ranucci M, Barbanti M, Grossi C *et al*: **Early and Midterm Outcome of Propensity-Matched Intermediate-Risk Patients Aged >80 Years With Aortic Stenosis Undergoing Surgical or Transcatheter Aortic Valve Replacement (from the Italian Multicenter OBSERVANT Study)**. *Am J Cardiol* 2016, **117**(9):1494-1501.

Rosato S, Santini F, Barbanti M, Biancari F, D'Errigo P, Onorati F, Tamburino C, Ranucci M, Covello RD, Santoro G *et al*: **Transcatheter Aortic Valve Implantation Compared With Surgical Aortic Valve Replacement in Low-Risk Patients**. *Circ* 2016, **9**(5):e003326.

**Long-term follow-up of previous published study: 5**

Kodali SK, Williams MR, Smith CR, Svensson LG, Webb JG, Makkar RR, Fontana GP, Dewey TM, Thourani VH, Pichard AD *et al*: **Two-year outcomes after transcatheter or surgical aortic-valve replacement**. *N Engl J Med* 2012, **366**(18):1686-1695.

Makkar RR, Fontana GP, Jilaihawi H, Kapadia S, Pichard AD, Douglas PS, Thourani VH, Babaliaros VC, Webb JG, Herrmann HC *et al*: **Transcatheter aortic-valve replacement for inoperable severe aortic stenosis.[Erratum appears in N Engl J Med. 2012 Aug 30;367(9):881]**. *N Engl J Med* 2012, **366**(18):1696-1704.

Kapadia SR, Leon MB, Makkar RR, Tuzcu EM, Svensson LG, Kodali S, Webb JG, Mack MJ, Douglas PS, Thourani VH *et al*: **5-year outcomes of transcatheter aortic valve replacement compared with standard treatment for patients with inoperable aortic stenosis (PARTNER 1): a randomised controlled trial**. *Lancet* 2015, **385**(9986):2485-2491.

Mack MJ, Leon MB, Smith CR, Miller DC, Moses JW, Tuzcu EM, Webb JG, Douglas PS, Anderson WN, Blackstone EH *et al*: **5-year outcomes of transcatheter aortic valve replacement or surgical aortic valve replacement for high surgical risk patients with aortic stenosis (PARTNER 1): a randomised controlled trial**. *Lancet* 2015, **385**(9986):2477-2484.

Sondergaard L, Steinbruchel DA, Ihlemann N, Nissen H, Kjeldsen BJ, Petursson P, Ngo AT, Olsen NT, Chang Y, Franzen OW *et al*: **Two-Year Outcomes in Patients With Severe Aortic Valve Stenosis Randomized to Transcatheter Versus Surgical Aortic Valve Replacement: The All-Comers Nordic Aortic Valve Intervention Randomized Clinical Trial**. *Circ* 2016, **9**(6).

**Ineligible follow-up: 6**

Brennan JM, Holmes DR, Sherwood MW, Edwards FH, Carroll JD, Grover FL, Tuzcu EM, Thourani V, Brindis RG, Shahian DM *et al*: **The Association of Transcatheter Aortic Valve Replacement Availability and Hospital Aortic Valve Replacement Volume and Mortality in the United States**. *Ann Thorac Surg* 2014, **98**(6):2016-2022.

Santarpino G, Pfeiffer S, Jessl J, Dell'Aquila A, Vogt F, von Wardenburg C, Schwab J, Sirch J, Pauschinger M, Fischlein T: **Clinical Outcome and Cost Analysis of Sutureless Versus Transcatheter Aortic Valve Implantation With Propensity Score Matching Analysis**. *Am J Cardiol* 2015, **116**(11):1737-1743.

Ailawadi G, LaPar DJ, Speir AM, Ghanta RK, Yarboro LT, Crosby IK, Lim DS, Quader MA, Rich JB: **Contemporary Costs Associated With Transcatheter Aortic Valve Replacement: A Propensity-Matched Cost Analysis**. *Ann Thorac Surg* 2016, **101**(1):154-160; discussion 160.

Biancari F, Barbanti M, Santarpino G, Deste W, Tamburino C, Gulino S, Imme S, Di Simone E, Todaro D, Pollari F *et al*: **Immediate outcome after sutureless versus transcatheter aortic valve replacement**. *Heart Vessels* 2016, **31**(3):427-433.

Mollmann H, Bestehorn K, Bestehorn M, Papoutsis K, Fleck E, Ertl G, Kuck KH, Hamm C: **In-hospital outcome of transcatheter vs. surgical aortic valve replacement in patients with aortic valve stenosis: complete dataset of patients treated in 2013 in Germany**. *Clin* 2016, **105**(6):553-559.

Zack CJ, Al-Qahtani F, Kawsara A, Al-Hijji M, Amin AH, Alkhouli M: **Comparative Outcomes of Surgical and Transcatheter Aortic Valve Replacement for Aortic Stenosis in Nonagenarians**. *Am J Cardiol* 2016.
