# Supplementary material for: Comparison of transcatheter aortic valve implantation with other approaches to treat aortic valve stenosis: a systematic review and meta-analysis
Source: Syst Rev. 2019 Feb 5;8:44. doi: 10.1186/s13643-019-0954-3 (PMC6362570; doi:10.1186/s13643-019-0954-3)
Supplement: Supplementary file 3 — Risk of bias assessment. (DOCX 22 kb) [file 13643_2019_954_MOESM3_ESM.docx]

**Additional file 3. Risk of bias assessment**

**Table A. Newcastle-Ottawa Quality Assessment Scale (NOS) for cohort studies**

| **Study Author** | **Year** | **Selection**  **Low = ≥ 3, Moderate = 2,**  **High = 0-1 points** | | | | **Comparability**  **Low = ≥ 2, Moderate = ≥ 1**  **High = 0 points** | **Outcome**  **Low = 3, Moderate = 2,**  **High = 0-1 points** | | | **Risk of bias** |
| --- | --- | --- | --- | --- | --- | --- | --- | --- | --- | --- |
|  |  | **Representativeness of the exposed cohort** | **Selection of the non exposed cohort** | **Ascertainment of exposure** | **Demonstration that outcome of interest was not present at start of study** | **Comparability of cohorts on the basis of the design or analysis** | **Assessment of outcome** | **Was follow-up long enough for outcomes to occur** | **Adequacy of follow up of cohorts** |  |
| Repossini | 2017 | 1 (b) | 1 (a) | 1 (a) | 1 (a) | 2 (a, b) | 1 (b) | 1 (a) | 1 (b) | Low |
| D'Onofrio | 2016 | 1 (b) | 0 (b) | 1 (a) | 1 (a) | 2 (a, b) | 1 (b) | 1 (a) | 1 (a) | Low |
| Kobrin | 2016 | 0 (c) | 1 (a) | 1 (a) | 1 (a) | 2 (a, b) | 1 (b) | 1 (a) | 1 (a) | Low |
| Hannan | 2016 | 1 (b) | 1 (a) | 1 (a) | 1 (a) | 1 (a) | 1 (b) | 1 (a) | 1 (a) | Moderate |
| Tamburiono | 2015 | 1 (b) | 1 (a) | 1 (a) | 1 (a) | 2 (a, b) | 1 (b) | 1 (a) | 1 (b) | Low |
| Schymik | 2015 | 1 (b) | 1 (a) | 1 (a) | 1 (a) | 2 (a, b) | 1 (b) | 1 (a) | 1 (a) | Low |
| Muneretto | 2015 | 1 (b) | 1 (a) | 1 (a) | 1 (a) | 2 (a, b) | 1 (b) | 1 (a) | 1 (b) | Low |
| Hoffmann | 2013 | 1 (b) | 1 (a) | 1 (a) | 1 (a) | 2 (a, b) | 1 (b) | 1 (a) | 1 (a) | Low |
| D'Onofrio | 2013 | 1 (b) | 1 (a) | 1 (a) | 1 (a) | 2 (a, b) | 1 (b) | 1 (a) | 1 (a) | Low |
| Piazza | 2013 | 1 (b) | 1 (a) | 1 (a) | 1 (a) | 2 (a, b) | 1 (b) | 1 (a) | 1 (b) | Low |
| Latib | 2012 | 1 (b) | 0 (b) | 1 (a) | 1 (a) | 2 (a, b) | 1 (b) | 1 (a) | 1 (a) | Low |
| Holzhey | 2012 | 1 (b) | 1 (a) | 1 (a) | 1 (a) | 2 (a, b) | 1 (b) | 1 (a) | 1 (a) | Low |
| Walther | 2010 | 1 (b) | 1 (a) | 1 (a) | 1 (a) | 2 (a, b) | 1 (b) | 1 (a) | 1 (a) | Low |

Letter in parenthesis refers to selected answer.

A study can be awarded a maximum of one point for each numbered item within the Selection and Outcome categories.

A maximum of two points can be given for Comparability.

**Table B. Cochrane risk of bias tool for randomized controlled trials**

| **Study**  **Author** | **Year** | **Trial name** | **Was randomization adequate?** | **Was allocation concealment adequate?** | **Were care providers masked?** | **Were patients masked?** | **Were outcome assessors masked?** | **Was overall attrition ≥20%?** | **Did the study use ITT analyses?** | **Selective reporting?** | **Other bias?** | **Risk of Bias** |
| --- | --- | --- | --- | --- | --- | --- | --- | --- | --- | --- | --- | --- |
| Reardon | 2017 | SURTAVI | Yes | Yes | No | No | Unclear | No | Yes | No | No | Low |
| Leon | 2016 | PARTNER 2A | Yes | Yes | No | No | Yes | No | Yes | No | No | Low |
| Thyregod | 2015 | NOTION | Yes | Yes | No | No | No | No | Yes | No | No | Low |
| Adams | 2014 | US CoreValve | Yes | Yes | No | No | Unclear | No | Yes | No | No | Low |
| Smith | 2011 | PARTNER A | Yes | Yes | No | No | Yes | No | Yes | No | No | Low |
| Leon | 2010 | PARTNER B | Yes | Yes | No | No | Yes | No | Yes | No | No | Low |

**Abbreviations:** ITT = Intention-to-treat analysis, NOTION = Nordic Aortic Valve Intervention, OBSERVANT = Observational Study of Effectiveness of SAVR-TAVR Procedures for Severe Aortic Stenosis Treatment, PARTNER = Placement of Aortic Transcatheter Valves, SAVR = Surgical aortic valve replacement, SURTAVI = Surgical Replacement and Transcatheter Aortic Valve Implantation
