# Supplementary material for: Comparison of transcatheter aortic valve implantation with other approaches to treat aortic valve stenosis: a systematic review and meta-analysis
Source: Syst Rev. 2019 Feb 5;8:44. doi: 10.1186/s13643-019-0954-3 (PMC6362570; doi:10.1186/s13643-019-0954-3)
Supplement: Supplementary file 4 — Patient characteristics and outcome in included studies. (DOCX 260 kb) [file 13643_2019_954_MOESM4_ESM.docx]

**Additional file 4. Patient characteristics and outcome in included studies**

**Table A. Patient characteristics in included studies**

| **Author, year** | **N** | **Age**  **years**  **Mean±SD** | **Female**  **N (%)** | **Log Euro**  **SCORE %**  **Mean±SD** | **STS risk**  **score %**  **Mean±SD** | **NYHA**  **III or IV**  **N (%)** | **LVEF**  **%**  **Mean±SD** | **AF**  **N (%)** | **DM**  **N (%)** | **CAD**  **N (%)** | **Prior MI**  **N (%)** | **PVD**  **N (%)** | **Prior**  **PPM**  **N (%)** |
| --- | --- | --- | --- | --- | --- | --- | --- | --- | --- | --- | --- | --- | --- |
| Reardon 2017 [1]  SURTAVI | TAVI:  879  SAVR:  867 | TAVI:  79.9±6.2  SAVR:  79.8±6.0 | TAVI:  371 (42.2)  SAVR:  383 (44.2) | TAVI:  11.9±7.6  SAVR:  11.6±8.0 | TAVI:  4.4±1.5  SAVR:  4.5±1.6 | TAVI:  529 (60.2)  SAVR:  500 (57.7) | NR | TAVI:  247 (28.1)  SAVR:  230 (26.5) | TAVI:  302 (34.4)  SAVR:  290 (33.4) | TAVI:  549 (62.5)  SAVR:  556 (64.1) | TAVI:  125 (14.2)  SAVR:  116 (13.4) | TAVI:  269 (30.6)  SAVR:  264 (30.4) | TAVI:  86 (9.8)  SAVR:  76 (8.8) |
| Leon 2016 [2]  PARTNER 2A | TAVI:  1011  SAVR:  1021 | TAVI:  81.5±6.7  SAVR:  81.7±6.7 | TAVI:  463 (45.8)  SAVR:  461 (45.2) | NR | TAVI:  5.8±2.1  SAVR:  5.8±1.9 | TAVI:  782/1011 (77.3)  SAVR:  776/1020 (76.1) | TAVI:  56.2±10.8  SAVR:  55.3±11.9 | TAVI:  313 (31.0)  SAVR:  359 (35.2) | TAVI:  381 (37.7)  SAVR:  349 (34.2) | TAVI:  700 (69.2)  SAVR:  679 (66.5) | TAVI:  185 (18.3)  SAVR:  179 (17.5) | TAVI:  282 (27.9)  SAVR:  336 (32.9) | TAVI:  118 (11.7)  SAVR:  123 (12.0) |
| Thyregod 2015 [3]  NOTION | TAVI:  145  SAVR:  135 | TAVI:  79.2±4.9  SAVR:  79.0±4.7 | TAVI:  67 (46.2)  SAVR:  64 (47.4) | TAVI:  8.4±4.0  SAVR:  8.9±5.5 | TAVI:  2.9±1.6  SAVR:  3.1±1.7 | TAVI:  70 (48.6)  SAVR:  61 (45.5) | NR | TAVI:  40/144 (27.8)  SAVR:  34/133 (25.6) | TAVI:  26 (17.9)  SAVR:  28 (20.7) | NR | TAVI:  8 (5.5)  SAVR:  6 (4.4) | TAVI:  6 (4.1)  SAVR:  9 (6.7) | TAVI:  5 (3.4)  SAVR:  6 (4.4) |
| Adams 2014 [4]  US CoreValve | TAVI:  394  SAVR:  401 | TAVI:  83.2±7.1  SAVR:  83.5±6.3 | TAVI:  183 (46.4)  SAVR:  189 (47.1) | TAVI:  17.6±13.0  SAVR:  18.4±12.8 | TAVI:  7.3±3.0  SAVR:  7.5±3.2 | TAVI:  338 (85.8)  SAVR:  348 (86.8) | NR | TAVI:  161/393 (41.0)  SAVR:  190/400 (47.5) | TAVI:  136 (34.5)  SAVR:  172 (42.9) | TAVI:  297 (75.4)  SAVR:  206/401 (76.3) | TAVI:  101 (25.6)  SAVR:  98 (24.4) | TAVI:  163/391 (41.7)  SAVR:  169/398 (42.5) | TAVI:  92 (23.4)  SAVR:  83 (20.7) |
| Smith 2011 [5]  PARTNER A | TAVI:  348  SAVR:  351 | TAVI:  83.6±6.8  SAVR:  84.5±6.4 | TAVI:  147/348 (42.2)  SAVR:  151/349 (43.3) | TAVI:  29.3±16.5  SAVR:  29.2±15.6 | TAVI:  11.8 ±3.3  SAVR:  11.7±3.5 | TAVI:  328/348 (94.3)  SAVR:  328/349 (94.0) | TAVI: 52.5±13.5  SAVR: 53.3±12.8 | TAVI:  80/196 (40.8)  SAVR:  73/171 (42.7) | NR | TAVI:  260/347 (74.9)  SAVR:  266/346 (76.9) | TAVI:  92/343 (26.8)  SAVR:  103/343(30.0) | TAVI:  148/344 (43.0)  SAVR:  142/341 (41.6) | TAVI:  69/345 (20)  SAVR:  76/347 (21.9) |
| Leon 2010 [6]  PARTNER B | TAVI:  179  SAVR:  179 | TAVI:  83.1±8.6  MT:  83.2±8.3 | TAVI:  97 (54.2)  MT:  95 (53.1) | TAVI:  26.4±17.2  MT:  30.4±19.1 | TAVI:  11.2±5.8  MT:  12.1±6.1 | TAVI:  165 (92.2)  MT:  168 (93.9) | TAVI: 53.9±13.1  MT: 51.1±14.3 | TAVI:  28/85 (32.9)  MT:  39/80 (48.8) | NR | TAVI:  121 (67.6)  MT:  133 (74.3) | TAVI:  33/177 (18.6)  MT:  47/178 (26.4) | TAVI:  54/178 (30.3)  MT:  45/179 (25.1) | TAVI:  35/153 (22.9)  MT:  31/159 (19.5) |
| Repossini 2017 [7] | TAVI:  142  SAVR:  142 | TAVI:  76.2±7.6  SAVR:  76.4±7.2 | TAVI:  57 (40.1)  SAVR:  54 (38.1) | NR | TAVI:  7.2±2.9  SAVR:  6.7±3.2 | TAVI:  67 (47.2)  SAVR:  69 (48.6) | TAVI:  52.9±14.1  SAVR:  53±12.9 | NR | TAVI:  41 (28.9)  SAVR:  43 (30.2) | NR | TAVI:  11 (7.7)  SAVR:  10 (7.0) | TAVI:  21 (14.8)  SAVR:  19 (13.3) | TAVI:  6 (4.2)  SAVR:  5 (3.5) |
| Hannan 2016 [8] | TAVI:  405  SAVR:  405 | NR | TAVI:  233 (57.5)  SAVR:  219 (54.1) | NR | NR | NR | NR | NR | TAVI:  135 (33.3)  SAVR:  145 (35.8.) | TAVI:  112 (27.7))  SAVR:  108 (26.7) | TAVI:  88 (21.7)  SAVR:  82 (20.3) | TAVI:  85 (21.0)  SAVR:  85 (21.0) | NR |
| D’Onofrio 2016  [9] | TAVI:  214  SAVR:  214 | TAVI:  77.7±7.94  SAVR:  77.4±5.4 | TAVI:  139 (65.0)  SAVR:  138 (64.5) | TAVI:  12.4±9.1  SAVR:  10.5±6.2 | NR | TAVI:  157 (73.4)  SAVR:  149 (69.6) | TAVI:  58.2±10.2  SAVR:  57.7±9.3 | NR | TAVI:  58 (27.1)  SAVR:  59 (27.6) | TAVI:  11 (5.1)  SAVR:  12 (5.6) | NR | TAVI:  48 (22.4)^c^  SAVR:  46 (21.5)^c^ | NR |
| Kobrin 2016  [10] | TAVI:  194  SAVR:  194 | TAVI:  77.7±0.9  SAVR:  78.0±0.5 | TAVI:  81 (41.8)  SAVR:  85 (43.8) | NR | NR | NR | NR | NR | TAVI:  133 (69)  SAVR:  128 (66) | NR | NR | TAVI:  167 (86)  SAVR:  170 (88) | NR |
| Tamburino 2015 [11]  OBSERVANT | TAVI:  650  SAVR:  650 | TAVI:  80.5 ± 6.2  SAVR:  80.3±5.1 | TAVI:  383 (58.9  SAVR:  387 (59.5) | TAVI:  9.5±7.1  SAVR:  10.2±9.2 | NR | TAVI:  385 (59.2)  SAVR:  388 (59.7) | TAVI:  53.6±11.4  SAVR:  54.2± 11.2 | NR | TAVI:  161 (24.8)  SAVR:  165 (25.4) | NR | TAVI:  72 (11.1)  SAVR:  75 (11.5) | TAVI:  124 (19.1)  SAVR:  126 (19.4) | NR |
| Schymik 2015 [12] | TAVI:  216  SAVR:  216 | TAVI:  78.3±5.2  SAVR:  78.2±4.6 | TAVI:  116^a^ (53.7)  SAVR:  105^a^ (48.6) | TAVI:  8.7±2.7  SAVR:  8.8±2.8 | NR | NR | TAVI:  62.2±11.3  SAVR:  62.0±10.5 | NR | NR | TAVI:  104 (48.1)  SAVR:  104 (48.1) | TAVI:^e^  5^a^ (2.3)  SAVR:^e^  7^a^ (3.2) | TAVI:  11 (5.1)  SAVR:  15 (6.9) | NR |
| Muneretto 2015 [13] | TAVI  204  SAVR  408 | TAVI:  80±2  SAVR: b  80±3  79±4 | TAVI:  91 (44.6)  SAVR:  98 (48)  105 (51.4) | TAVI:  19.5±6.7  SAVR:  19.2±7.4  18.9±5.9 | SAVI:  8.2±4.2  SAVR:  8.3±4.4  7.9±3.2 | TAVI:  137 (67.1)  SAVR:  125 (61.2)  130 (64) | TAVI:  54.6±6.8  SAVR:  54.7±5.1  55.1±7.3 | NR | TAVI:  62 (30.3)  SAVR:  54 (26.4)  57 (28) | TAVI:  53 (25.9)  SAVR:  49 (24.0)  42 (20.6) | TAVI:  15 (7.5)  SAVR:  17 (8.5)  12 (6) | TAVI:  43 (21)  SAVR:  46 (22.6)  40 (19.6) | NR |
| Hoffmann 2013 [14] | TAVI:  135  MT:  135 | TAVI:  80±6  MT:  79±6 | TAVI:  77 (57.0)  MT:  75 (55.6) | TAVI:  21±13  MT:  21±19 | NR | NR | NR | NR | TAVI:  38 (28)  MT:  46 (34) | TAVI:  65 (47)  MT:  66 (48) | TAVI**:** ^d^  7 (5)  MT: ^d^  6 (4) | NR | NR |
| D’Onofrio 2013 [15] | TAVI:  143  SAVR:  143 | TAVI:  77.6±9  SAVR:  73.5±12.6 | TAVI:  90^a^ (62.9)  SAVR:  72^a^ (50.3) | TAVI:  20.2±12.5  SAVR:  18.3±14.6 | NR | TAVI:  93^a^ (65)  SAVR:  78^a^ (54.5) | TAVI:  56.1±13  SAVR:  58.1±10.9 | NR | NR | NR | NR | TAVI:  61^a^ (42.7)  SAVR:  53^a^ (37.1) | NR |
| Piazza 2013 [16] | TAVI:  405  SAVR  405 | TAVI:  79.9±6.0  SAVR  79.4±4.8 | TAVI:  230 (56.8)  SAVR  226 (55.8) | TAVI:  17.1±10.7  SAVR  17.5±12.1 | NR | TAVI:  305 (87.1)  SAVR  304 (86.8) | NR | NR | TAVI:  111 (27.4)  SAVR  98 (25.7) | TAVI:  94 (56.6)  SAVR  96 (57.8) | NR | TAVI:  33 (8.2)  SAVR  41 (10.1) | NR |
| Latib 2012 [17] | TAVI:  111  SAVR:  111 | TAVI:  80.5 ± 6.9  SAVR:  79.4 ± 3.0 | NR | TAVI:  23.2 ± 15.1  SAVR:  24.4 ± 13.4 | TAVI:  4.57 ± 2.28  SAVR:  4.60 ± 2.63 | TAVI:  75 (67.6)  SAVR:  77 (69.4) | TAVI:  53.5 ± 12.5  SAVR:  53.6 ± 10.7 | NR | TAVI:  21 (18.9)  SAVR:  24 (21.6) | TAVI:  44 (39.6)  SAVR:  51 (45.9) | TAVI:  16 (14.4)  SAVR:  16 (14.4) | TAVI:  29 (26.1)  SAVR:  38 (34.2) | NR |
| Holzhey 2012 [18] | TAVI:  167  SAVR:  167 | TAVI:  79.8±5.4  SAVR:  80.5±4.6 | TAVI:  108 (64.7)  SAVR:  108 (64.7) | TAVI:  18.7±11.1  SAVR:  18.3±14.0 | NR | NR | TAVI:  56.0±14.7  SAVR:  56.3±14.1 | NR | TAVI:  66 (39.5)  SAVR:  74 (44.3) | NR | NR | TAVI:  46 (27.5)  SAVR:  43 (25.7) | NR |
| Walther 2010 [19] | TAVI:  100  SAVR:  100 | TAVI:  82.7±5  SAVR:  82.4±4 | TAVI:  77 (77)  SAVR:  70 (70) | TAVI:  29±13  SAVR:  30±13 | TAVI:  15.2±8.3  SAVR:  NR | TAVI:  100 (100)  SAVR:  NR | TAVI:  54.0±15  SAVR:  56.3±18 | NR | TAVI:  40 (40)  SAVR:  37 (37) | NR | NR | NR | NR |

**Abbreviations:** AF = Atrial fibrillation; CAD = Coronary artery disease; DM = Diabetes mellitus; Log euroSCORE = Logistic European System for Cardiac Operative Risk Evaluation; LVEF = Left ventricular ejection fraction; MI = Myocardial infarction; NYHA = New York Heart Association; N = Number of patients; NOTION = Nordic Aortic Valve Intervention; NR = Not reported; OBSERVANT = Observational Study of Effectiveness of SAVR-TAVR Procedures for Severe Aortic Stenosis Treatment; PARTNER = Placement of Aortic Transcatheter Valves; PPM = Permanent Pacemaker; PVD = Peripheral vascular disease, STS = Score Society of Thoracic Surgeons; SAVR = Surgical aortic valve replacement; SUAVR = Sutureless surgical aortic valve replacement; SURTAVI = Surgical Replacement and Transcatheter Aortic Valve Implantation ;TAVI = Transcatheter aortic valve replacement

a, Self-calculated from percentage

b, Baseline characteristics reported separately for convention and sutureless surgical aortic valve replacement

c, Reported as extracardiac arteriopathy

d, Reported as MI <90 days

,

**Table B. Outcome observed in included studies**

| **Study,**  **Author, year** | **ITT**  **N** | **All-cause**  **death**  **N (%)** | **Stroke**  **N (%)** | **TIA**  **N (%)** | **MI**  **N (%)** | **Major**  **Bleeding**  **N (%)** | **Major**  **vascular complications**  **N (%)** | **Moderate or severe PVR**  **N (%)** | **New PPM**  **N (%)** | **Endpoint definitions**  **VARC or VARC-2** |
| --- | --- | --- | --- | --- | --- | --- | --- | --- | --- | --- |
| Reardon 2017 [1]  SURTAVI | TAVI:  879  SAVR:  867 | *30 days:^l,r^*  TAVI:  18/879 (2.0)  SAVR:  11/867 (1.3)  *1 year:^l,r^*  TAVI:  62/879 (7.0)  SAVR:  59/867 (6.8) | *30 days:^l,r^*  TAVI:  23 (2.6)  SAVR:  42 (4.8)  *1 year:^l,r^*  TAVI:  48 (5.5)  SAVR:  59 (6.8) | *30 days:^l,r^*  TAVI:  8 (0.9)  SAVR:  6 (0.7)  *1 year:^l,r^*  TAVI:  30 (3.4)  SAVR:  17 (2.0) | *30 days:^l,r^*  TAVI:  8 (0.9)  SAVR:  6 (0.7)  *1 year:^l,r^*  TAVI:  18 (2.0)  SAVR:  15 (1.7) | *30 days: ^j,l,n^*  TAVI:  107 (12.2)  SAVR:  81 (9.3)  *1 year:*  NR | *30 days:^l,n^*  TAVI:  53 (6.0)  SAVR:  10 (1.1)  *1 year:*  NR | *30 days:^r,t^*  TAVI:  28/820 (3.4)  SAVR:  2/675 (0.3)  *1 year: ^r^*  TAVI:  31/580 (5.3)  SAVR:  3/488 (0.6) | *30 days:^l,n^*  TAVI:  228 (25.9)  SAVR:  57 (6.6)  *1 year:*  NR | VARC-2 |
| Leon 2016 [2]  PARTNER 2A | TAVI:  1011  SAVR:  1021 | *30 days:^a^*  TAVI:  39/1011 (3.9)  SAVR:  41/1021 (4.1)  *1 year:^a^*  TAVI:  123/1011 (12.3)  SAVR:  124/1021 (12.9) | *30 days:^a^*  TAVI:  55 (5.5)  SAVR:  61 (6.1)  *1 year:^a^*  TAVI:  78 (8.0)  SAVR:  79 (8.1) | *30 days:^a^*  TAVI:  9 (0.9)  SAVR:  4 (0.4)  *1 year:^a^*  TAVI:  23 (2.4)  SAVR:  16 (1.8) | *30 days:^a^*  TAVI:  12 (1.2)  SAVR:  19 (1.9)  *1 year :^a^*  TAVI:  24 (2.5)  SAVR:  29 (3.0) | *30 days: ^a,q^*  TAVI:  105 (10.4)  SAVR:  442 (43.4)  *1 year:^a,q^*  TAVI:  151 (15.2)  SAVR:  460 (45.5) | *30 days :^a^*  TAVI:  80 (7.9)  SAVR:  51 (5.0)  *1 year:^a^*  TAVI:  84 (8.4)  SAVR:  54 (5.3) | *30 days:^r^*  TAVI:  33/872 (3.7)  SAVR:  4/757 (0.6)  *1 year:^r^*  TAVI:  25/728 (3.4)  SAVR:  2/611 (0.4) | *30 days:^a^*  TAVI:  85 (8.5)  SAVR:  68 (6.9)  *1 year:^a^*  TAVI:  98 (9.9)  SAVR:  85 (8.9) | VARC and VARC-2 |
| Thyregod 2015 [3]  NOTION | TAVI:  145  SAVR:  135 | *30 days:^a,b^*  TAVI:  3/142 (2.1)  SAVR:  5/134 (3.7)  *1 year:^a,b^*  TAVI:  7/142 (4.9)  SAVR:  10/134 (7.5) | *30 days:^a,b^*  TAVI:  2 (1.4)  SAVR:  4 (3.0)  *1 year:^a,b^*  TAVI:  4 (2.9)  SAVR:  6 (4.6) | *30 days:^a,b^*  TAVI:  2 (1.4)  SAVR:  0  1 year*:^a,b^*  TAVI:  2 (2.1)  SAVR:  2 (1.6) | *30 days:^a,b^*  TAVI:  4 (2.8)  SAVR:  8 (6.0)  *1 year:^a,b^*  TAVI:  5 (3.5)  SAVR:  8 (6.0) | *30 days:^b,e,j^*  TAVI:  16 (11.3)  SAVR:  28 (20.9)  *1 year:*  NR | *30 days: ^b,e^*  TAVI:  8 (5.6)  SAVR:  2 (1.5)  *1 year:*  NR | *30 days:*  NR  *1 year:^l^*  TAVI:  19/121 (15.7)  SAVR:  1/113 (0.9) | *30 days:^a,b^*  TAVI:  46 (34.1)  SAVR:  2 (1.6)  *1 year:^a,b^*  TAVI:  51 (38.0)  SAVR:  3 (2.4) | VARC |
| Adams 2014 [4]  US CoreValve | TAVI:  394  SAVR:  401 | *30 days:^a,b^*  TAVI:  13/390 (3.3)  SAVR:  16/357 (4.5)  *1 year:^a,b^*  TAVI:  55/390 (14.2)  SAVR:  67/357 (19.1) | *30 days:^a,b^*  TAVI:  19 (4.9)  SAVR:  22 (6.2)  *1 year:^a,b^*  TAVI:  33 (8.8)  SAVR:  42 (12.6) | *30 days:^a,b^*  TAVI:  3 (0.8)  SAVR:  1 (0.3)  *1 year:^a,b^*  TAVI:  6 (1.6)  SAVR:  5 (1.6) | *30 days:^a,b^*  TAVI:  3 (0.8)  SAVR:  3 (0.8)  *1 year:^a,b^*  TAVI:  7 (1.9)  SAVR:  5 (1.5) | *30 days:^a,b^*  TAVI:  109 (28.1)  SAVR:  123 (34.5)  *1 year:^a,b^*  TAVI:  114 (29.5)  SAVR:  130 (36.7) | *30 days:^a,b^*  TAVI:  23 (5.9)  SAVR:  6 (1.7)  *1 year:^a,b^*  TAVI:  24 (6.2)  SAVR:  7 (2.0) | *30 days:^r^*  TAVI:  32/365 (9.0)  SAVR:  3/317 (1.0)  *1 year: ^r^*  TAVI:  18/299 (6.1)  SAVR:  1/228 (0.5) | *30 days:^a,b^*  TAVI:  76 (19.8)  SAVR:  25 (7.1)  *1 year:^a,b^*  TAVI:  85 (22.3)  SAVR:  38 (11.3) | VARC |
| Smith 2011 [5]  PARTNER A | TAVI:  348  SAVR:  351 | *30 days:^a^*  TAVI:  12/348 (3.4)  SAVR:  22/351 (6.5)  *1 year:^a^*  TAVI:  84/348 (24.2)  SAVR:  89/351 (26.8) | *30 days:^a,k^*  TAVI:  16 (4.7)  SAVR:  8 (2.4)  *1 year:^a,k^*  TAVI:  20 (6.0)  SAVR:  10 (3.1) | *30 days:^a^*  TAVI:  3 (0.9)  SAVR:  1 (0.3)  *1 year:^a^*  TAVI:  7 (2.3)  SAVR  4 (1.5) | *30 days:^a^*  TAVI:  0  SAVR:  2 (0.6)  *1 year:^a^*  TAVI:  1 (0.4)  SAVR:  2 (0.6) | *30 days:^a^*  TAVI:  32 (9.3)  SAVR:  67 (19.5)  *1 year:^a^*  TAVI:  49 (14.7)  SAVR:  85 (25.7) | *30 days:^a^*  TAVI:  38 (11.0)  SAVR:  11 (3.2)  *1 year:^a^*  TAVI:  39 (11.3)  SAVR:  12 (3.5) | *30 days:^r^*  TAVI:  35/287 (12.2)  SAVR:  2/229 (0.9)  *1 year:^r^*  TAVI:  15/222 (6.8)  SAVR:  3/159 (1.9) | *30 days:^a^*  TAVI:  13 (3.8)  SAVR:  12 (3.6)  *1 year:^a^*  TAVI:  19 (5.7)  SAVR:  16 (5.0) | Modified VARC for vascular compli-  cations |
| Leon 2010 [6]  PARTNER B | TAVI:  179  MT:  179 | *30 days:*  TAVI:  9/179 (5.0)  MT:  5/179 (2.8)  *1 year:*  TAVI:  55/179 (30.7)  MT:  89/179 (49.7) | *30 days:*  TAVI:  12 (6.7)  MT:  3 (1.7)  *1 year:*  TAVI:  18 (10.0)  MT:  8 (4.5) | *30 days:*  TAVI:  0  MT:  0  *1 year:*  TAVI:  1 (0.6)  MT:  0 | *30 days:*  TAVI:  0  MT:  0  *1 year:*  TAVI:  1 (0.6)  MT:  1 (0.6) | *30 days:*  TAVI:  30 (16.8)  MT:  7 (3.9)  *1 year:*  TAVI:  40 (22.3)  MT:  20 (11.2) | *30 days*:  TAVI:  29 (16.2)  MT:  2 (1.1)  *1 year:*  TAVI:  30 (16.8)  MT:  4 (2.2) | *30 days:^r^*  TAVI:  18/153 (12)  MT:  0/125 (0)  *1 year:^r^*  TAVI:  11/98 (11)  MT:  0/52 (0) | *30 days:*  TAVI:  6 (3.4)  MT:  9 (5.0)  *1 year:*  TAVI:  8 (4.5)  MT:  14 (7.8) | Modified VARC for vascular compli-  cations |
| D’Onofrio 2016 [9] | TAVI:  214  SAVR:  214 | *30 days:*  TAVI:  8/214 (3.7)  SAVR:  5/214 (2.3)  *1 year:*  TAVI:  20/214 (9.4)  SAVR:  12/214 (5.8) | *30 days:*  TAVI:  4 (1.9)  SAVR:  4 (1.9)  *1 year:*  NR | NR | *30 days:*  TAVI:  2 (0.9)  SAVR:  1 (0.5)  *1 year:*  NR | *30 days:^j^*  TAVI:  34 (16.1)  SAVR:  40 (18.7)  *1 year:*  NR | NR | *30 days:*  TAVI:  11 (5.3)  SAVR:  1 (0.5)  *1 year:*  NR | *30 days:*  TAVI:  6 (2.8)  SAVR:  20 (9.4)  *1 year:*  NR | VARC |
| Repossini 2017 [7] | TAVI:  142  SAVR:  142 | *30 days:*  TAVI:  9/142 (6.3)  SAVR:  3/142 (2.1) | *30 days:^i^*  TAVI:  4 (2.8)  SAVR:  2 (1.4) | NR | *30 days:^p^*  TAVI:  0  SAVR:  0 | NR | *30 days:*  TAVI:  10 (7.4)  SAVR:  0 | *30 days:*  TAVI:  5 (3.5)  SAVR:  1 (0.7) | *30 days:*  TAVI:  17 (12.0)  SAVR:  1 (0.7) | VARC-2 |
| Hannan 2016 [8] | TAVI:  405  SAVR:  405 | *30 days:^,l^*  TAVI:  19/405 (4.7)  SAVR:  19/405 (4.7)  *1 year:^a^*  TAVI:  63/405 (15.6)  SAVR:  53/405 (13.1) | NR | NR | NR | NR | NR | NR | NR | - |
| Kobrin 2016 [10] | TAVI:  194  SAVR:  194 | *30 days:*  TAVI:  20/194 (10)  SAVR:  15/194 (8) | *30 days:^i^*  TAVI:  3 (2)  SAVR:  8 (4) | NR | *30 days:*  TAVI:  2 (1)  SAVR:  1 (1) | NR | NR | NR | *30 days:*  TAVI:  18 (9)  SAVR:  15 (8) | - |
| Tamburino 2015 [11]  OBSERVANT | TAVI:  650  SAVR:  650 | *30 days:*  TAVI:  20/650 (3.2)  SAVR:  24/650 (3.8)  *1 year:^a^*  TAVI:  83/650 (13.8)  SAVR:  82/650 (13.6) | *30 days:^f^*  TAVI:  8 (1.3)  SAVR:  14 (2.2)  *1 year:^g^*  TAVI:  37 (6.4)  SAVR:  29 (4.9) | NR | *30 days:^f^*  TAVI:  3 (0.5)  SAVR:  5 (0.8)  *1 year:^g^*  TAVI:  15 (3.1)  SAVR:  18 (3.8) | NR | *30 days:^f^*  TAVI:  48 (7.9)  SAVR:  3 (0.5)  *1 year:*  NR | *30 days:^f^*^,l^  TAVI:  64 (9.8)  SAVR:  13 (2.0)  *1 year:*  NR | *30 days:^f^*  TAVI:  98 (15.5)  SAVR:  23 (3.6)  *1 year:^g^*  TAVI:  114 (18.5)  SAVR:  43 (7.3) | VARC  for PVR |
| Schymik 2015 [12] | TAVI:  216  SAVR:  216 | *30 days:****^l^***  TAVI:  3/216 (1.4)  SAVR:  9/216 (4.2)  *1 year:****^l,w^***  TAVI:  25/216 (11.6)  SAVR:  16/216 (7.4) | *30 days:^k,l^*  TAVI:  3 (1.4)  SAVR:  2 (1.0)  *1 year:*  NR | *30 days:*  TAVI:  0  SAVR:  0  *1 year:*  NR | *30 days:^l^*  TAVI:  1 (0.5)  SAVR:  2 (0.9)  *1 year:*  NR | *30 days:^l^*  TAVI:  9 (4.2)  SAVR:  45 (20.8)  *1 year:*  NR | *30 days:^l^*  TAVI:  23 (10.6)  SAVR:  0  *1 year:*  NR | NR | *30 days:^l^*  TAVI:  30 (13.9)  SAVR:  10 (4.6)  *1 year:*  NR | VARC-2 |
| Muneretto 2015 [13] | TAVI:  204  SAVR:  408 | *30 days:^o^*  TAVI:  20/204 (9.8)  SAVR:  19/408 (4.7) | *30 days:^p,o^*  TAVI:  7 (3.4)  SAVR:  10 (2.5) | NR | NR | *30 days:^p,h,o^*  TAVI:  4 (1.9)  SAVR:  16 (3.9) | *30 days:^p,d,o^*  TAVI:  20 (9.8)  SAVR:  0 | *30 days:^p,v,o^*  TAVI:  18 (8.8)  SAVR:  5 (1.2) | *30 days:^p^*  TAVI:  30 (14.7)  SAVR:  28 (6.9) | - |
| Hoffmann 2013 [14] | TAVI:  135  MT:  135 | *30 days:*  TAVI:  16/135 (12)  MT:  10/135 (7)  *1 year:*  TAVI:  28/135 (21)  MT:  69/135 (51)^u^ | NR | NR | NR | NR | NR | NR | *30 days:*  TAVI:  19 (14.1)  MT:  2 (1.5)  *1 year:*  NR | - |
| D’Onofrio 2013 [15] | TAVI:  143  SAVR:  143 | *30 days:*^o^  TAVI:  10/143 (7)  SAVR:  1/143 (0.7) | *30 days:*^o,^*^p^*  TAVI:  4 (2.8)  SAVR:  0 | NR | *30 days:*^o,^*^p^*  TAVI:  5 (3.5)  SAVR:  1 (0.7)^o^ | NR | NR | NR | *30 days:*^o,^*^p^*  TAVI:  7 (4.9)  SAVR:  2 (1.4)^o^ | VARC |
| Piazza 2013 [16] | TAVI:  405  SAVR  405 | *30 days:*  TAVI:  33/405 (8.1)  SAVR:  25/405 (6.2)  *1 year:*  TAVI:  71/405 (17.5)  SAVR:  67/405 (16.5) | NR | NR | NR | NR | NR | NR | NR | - |
| Latib 2012 [17] | TAVI:  111  SAVR:  111 | *30 days:*  TAVI:  2/111 (1.8)  SAVR:  2/111 (1.8)  *1 year:*  TAVI:  7/111 (6.4)  SAVR:  9/111 (8.1) | *30 days:*  TAVI:  1 (0.9)  SAVR:  2 (1.8)  *1 year:*  TAVI:  1 (0.9)  SAVR:  3 (2.9) | *30 days:*  TAVI:  3 (2.7)  SAVR:  7 (6.4)  *1 year:*  TAVI:  4 (3.7  SAVR:  7 (6.4) | *30 days:*  TAVI:  0  SAVR:  2 (2.7)  *1 year:*  TAVI:  0  SAVR:  2 (2.7) | *30 days:^c^*  TAVI:  43 (38.7)  SAVR:  63 (56.8)  *1 year:*  NR | *30 days:^c^*  TAVI:  16 (14.4)  SAVR:  0  *1 year:*  NR | NR | *30 days:^c^*  TAVI:  13 (11.7)  SAVR:  3 (2.7)  *1 year:*  NR | VARC |
| Holzhey 2012 [18] | TAVI:  167  SAVR:  167 | *30 days:*  TAVI:  14/167 (8.4)  SAVR:  18/167 (10.8) | *30 days:^p^*  TAVI:  1 (0.6)  SAVR:  3 (1.8) | *30 days:^p,s^*  TAVI:  4 (2.4)  SAVR:  7 (4.2) | NR | NR | NR | NR | *30 days:^p^*  TAVI:  25 (15)  SAVR:  10 (6.0) | - |
| Walther 2010 [19] | TAVI:  100  SAVR:  100 | *30 days:*  TAVI:  10/100  SAVR:  15/100  *1 year:*  TAVI:  28/100  SAVR:  31/100 | *30 days:^p^*  TAVI:  0  SAVR:  2 (2)  *1 year:*  NR | NR | NR | NR | NR | NR | *30 days:^p^*  TAVI:  9 (10.1)  SAVR:  NR  *1 year:*  NR | - |

Abbreviations: ITT = Intention-to-treat; MI = Myocardial infarction; N = Number of patients; NOTION = Nordic Aortic Valve Intervention; NR = Not reported; OBSERVANT = Observational Study of Effectiveness of SAVR-TAVR Procedures for Severe Aortic Stenosis Treatment; PAR = Paravalvular aortic regurgitation; PARTNER = Placement of Aortic Transcatheter Valves ; PPM = Permanent Pacemaker; SAVR = Surgical aortic valve replacement; SUAVR = Sutureless surgical aortic valve replacement; SURTAVI = Surgical Replacement and Transcatheter Aortic Valve Implantation; TAVI = Transcatheter aortic valve replacement, TIA =Transient ischemic attack; VARC = Valve Academic Research Consortium

a, Percentages are from Kaplan-Meier analysis

b, As-treated population

c, Reported as periprocedural and in-hospital outcome

d, Type not specified

e, Reported as events during index-hospitalization or 30 days

f, Reported as periprocedural

g, Competitive risk estimates

h, Reported as bleeding requiring revisions

i, Reported as cerebrovascular accident

j, Reported as major or life-threatening bleeding

k, Major and minor stroke

l, Events self-calculated from percentage

m, Reported as postoperative neurologic complications

n, Modified intention-to-treat population

o, Events from sutureless and conventional SAVR added and percentages self-calculated

p, Reported as postoperative

q, Life-threatening or disabling bleeding

r, Provided in the supplement

s, Reported as cerebral ischemia

t, Reported at discharge

u, Self-calculated percentage (41% reported in the publication)

v, Aortic regurgitation greater than grade 2

w, Percentages extracted from figure and self-calculated

**References**

[1] Reardon MJ, Van Mieghem NM, Popma JJ, Kleiman NS, Sondergaard L, Mumtaz M, et al. Surgical or Transcatheter Aortic-Valve Replacement in Intermediate-Risk Patients. New England Journal of Medicine. 2017 04 06;376(14):1321-31.

[2] Leon MB, Smith CR, Mack MJ, Makkar RR, Svensson LG, Kodali SK, et al. Transcatheter or Surgical Aortic-Valve Replacement in Intermediate-Risk Patients. New England Journal of Medicine. 2016 Apr 28;374(17):1609-20.

[3] Thyregod HG, Steinbruchel DA, Ihlemann N, Nissen H, Kjeldsen BJ, Petursson P, et al. Transcatheter Versus Surgical Aortic Valve Replacement in Patients With Severe Aortic Valve Stenosis: 1-Year Results From the All-Comers NOTION Randomized Clinical Trial. J Am Coll Cardiol. 2015 May 26;65(20):2184-94.

[4] Adams DH, Popma JJ, Reardon MJ, Yakubov SJ, Coselli JS, Deeb GM, et al. Transcatheter aortic-valve replacement with a self-expanding prosthesis. N Engl J Med. 2014 May 8;370(19):1790-8.

[5] Smith CR, Leon MB, Mack MJ, Miller DC, Moses JW, Svensson LG, et al. Transcatheter versus surgical aortic-valve replacement in high-risk patients. N Engl J Med. 2011 Jun 9;364(23):2187-98.

[6] Leon MB, Smith CR, Mack M, Miller DC, Moses JW, Svensson LG, et al. Transcatheter aortic-valve implantation for aortic stenosis in patients who cannot undergo surgery. N Engl J Med. 2010 Oct 21;363(17):1597-607.

[7] Repossini A, Di Bacco L, Passaretti B, Grubitzsch H, Schäfer C, Claus B, et al. Early hemodynamics and clinical outcomes of isolated aortic valve replacement with stentless or transcatheter valve in intermediate-risk patients. Journal of Thoracic and Cardiovascular Surgery. 2017;153(3):549-58.e3.

[8] Hannan EL, Samadashvili Z, Stamato NJ, Lahey SJ, Wechsler A, Jordan D, et al. Utilization and 1-Year Mortality for Transcatheter Aortic Valve Replacement and Surgical Aortic Valve Replacement in New York Patients With Aortic Stenosis: 2011 to 2012. Jacc: Cardiovascular Interventions. 2016 Mar 28;9(6):578-85.

[9] D'Onofrio A, Salizzoni S, Rubino AS, Besola L, Filippini C, Alfieri O, et al. The rise of new technologies for aortic valve stenosis: A comparison of sutureless and transcatheter aortic valve implantation. Journal of Thoracic & Cardiovascular Surgery. 2016 Jul;152(1):99-109.e2.

[10] Kobrin DM, McCarthy FH, Herrmann HC, Anwaruddin S, Kobrin S, Szeto WY, et al. Transcatheter and Surgical Aortic Valve Replacement in Dialysis Patients: A Propensity-Matched Comparison. Annals of Thoracic Surgery. 2015 Oct;100(4):1230-6; discussion 6-7.

[11] Tamburino C, Barbanti M, D'Errigo P, Ranucci M, Onorati F, Covello RD, et al. 1-Year Outcomes After Transfemoral Transcatheter or Surgical Aortic Valve Replacement: Results From the Italian OBSERVANT Study. Journal of the American College of Cardiology. 2015 Aug 18;66(7):804-12.

[12] Schymik G, Heimeshoff M, Bramlage P, Herbinger T, Wurth A, Pilz L, et al. A comparison of transcatheter aortic valve implantation and surgical aortic valve replacement in 1,141 patients with severe symptomatic aortic stenosis and less than high risk. Catheterization & Cardiovascular Interventions. 2015 Oct;86(4):738-44.

[13] Muneretto C, Alfieri O, Cesana BM, Bisleri G, De Bonis M, Di Bartolomeo R, et al. A comparison of conventional surgery, transcatheter aortic valve replacement, and sutureless valves in "real-world" patients with aortic stenosis and intermediate- to high-risk profile. Journal of Thoracic & Cardiovascular Surgery. 2015 Dec;150(6):1570-7; discussion 7-9.

[14] Hoffmann R, Almutairi B, Herpertz R, Lotfipour S, Stohr R, Aktug O, et al. Two-year mortality after transcatheter aortic valve implantation versus medical therapy for high-surgical risk or inoperable aortic stenosis patients. Journal of Heart Valve Disease. 2013 Jan;22(1):71-8.

[15] D'Onofrio A, Rizzoli G, Messina A, Alfieri O, Lorusso R, Salizzoni S, et al. Conventional surgery, sutureless valves, and transapical aortic valve replacement: what is the best option for patients with aortic valve stenosis? A multicenter, propensity-matched analysis. Journal of Thoracic & Cardiovascular Surgery. 2013 Nov;146(5):1065-70; discussion 70-1.

[16] Piazza N, Kalesan B, van Mieghem N, Head S, Wenaweser P, Carrel TP, et al. A 3-center comparison of 1-year mortality outcomes between transcatheter aortic valve implantation and surgical aortic valve replacement on the basis of propensity score matching among intermediate-risk surgical patients. Jacc: Cardiovascular Interventions. 2013 May;6(5):443-51.

[17] Latib A, Maisano F, Bertoldi L, Giacomini A, Shannon J, Cioni M, et al. Transcatheter vs surgical aortic valve replacement in intermediate-surgical-risk patients with aortic stenosis: a propensity score-matched case-control study. American Heart Journal. 2012 Dec;164(6):910-7.

[18] Holzhey DM, Shi W, Rastan A, Borger MA, Hansig M, Mohr FW. Transapical versus conventional aortic valve replacement--a propensity-matched comparison. Heart Surg Forum. 2012 Feb;15(1):E4-8.

[19] Walther T, Schuler G, Borger MA, Kempfert J, Seeburger J, Ruckert Y, et al. Transapical aortic valve implantation in 100 consecutive patients: comparison to propensity-matched conventional aortic valve replacement. European Heart Journal. 2010 Jun;31(11):1398-403.
