# Supplementary material for: Comparison of transcatheter aortic valve implantation with other approaches to treat aortic valve stenosis: a systematic review and meta-analysis
Source: Syst Rev. 2019 Feb 5;8:44. doi: 10.1186/s13643-019-0954-3 (PMC6362570; doi:10.1186/s13643-019-0954-3)
Supplement: Supplementary file 5 — Funnel plots and Egger’s test. (DOCX 37 kb) [file 13643_2019_954_MOESM5_ESM.docx]

**Additional file 5. Funnel plots and Egger’s test for small study effects**

**Figure A. Mortality 30 days TAVI versus SAVR**

**Abbreviations:** CI = Confidence interval; RCT = Randomized controlled trial; SAVR = Surgical aortic valve replacement; TAVI = Transcatheter-aoritc valve replacment

####

**Figure B. Mortality 1 year TAVI versus SAVR**

**Abbreviations:** CI = Confidence interval; RCT = Randomized controlled trial; SAVR = Surgical aortic valve replacement; TAVI = Transcatheter-aoritc valve replacment
