# Supplementary material for: Comparison of transcatheter aortic valve implantation with other approaches to treat aortic valve stenosis: a systematic review and meta-analysis
Source: Syst Rev. 2019 Feb 5;8:44. doi: 10.1186/s13643-019-0954-3 (PMC6362570; doi:10.1186/s13643-019-0954-3)
Supplement: Supplementary file 6 — Summary of findings tables. (DOCX 24 kb) [file 13643_2019_954_MOESM6_ESM.docx]

**Additional file 6. Summary of findings tables**

**Table A. Summary of findings regarding mortality TAVI versus SAVR**

| **Certainty assessment** | | | | | | | **№ of patients** | | **Effect** | | **Certainty** |  |
| --- | --- | --- | --- | --- | --- | --- | --- | --- | --- | --- | --- | --- |
| **№ of studies** | **Study design** | **Risk of bias** | **Inconsistency** | **Indirectness** | **Imprecision** | **Other considerations** | **TAVI** | **SAVR** | **Relative (95% CI)** | **Absolute (95% CI)** |  |  |
| **Mortality 30 days** | | | | | | | | | | | | |
| 5 | RCTs | not serious | not serious | not serious | serious ^a,b^ | none | 85/2773 (3.1%) | 95/2731 (3.5%) | **RR 0.87** (0.61 to 1.23) | **5 fewer per 1.000** (from 8 more to 14 fewer) | ⨁⨁⨁◯ MODERATE |  |
| **Mortality 30 days** | | | | | | | | | | | | |
| 12 | Propensity-matched studies | not serious | not serious | not serious | serious ^b^ | none | 168/2951 (5.7%) | 155/3155 (4.9%) | **RR 1.16** (0.85 to 1.58) | **8 more per 1.000** (from 7 fewer to 28 more) | ⨁◯◯◯ VERY LOW |  |
| **Mortality 1 year** | | | | | | | | | | | | |
| 5 | RCTs | not serious | not serious | not serious | not serious | none | 331/2773 (11.9%) | 349/2731 (12.8%) | **RR 0.93** (0.81 to 1.07) | **9 fewer per 1.000** (from 9 more to 24 fewer) | ⨁⨁⨁⨁ HIGH |  |
| **Mortality 1 year** | | | | | | | | | | | | |
| 8 | Propensity-matched studies | not serious | not serious | not serious | serious ^b^ | none | 347/2268 (15.3%) | 307/2268 (13.5%) | **RR 1.13** (0.98 to 1.30) | **18 more per 1.000** (from 3 fewer to 41 more) | ⨁◯◯◯ VERY LOW |  |

**Abbreviations:** CI = Confidence interval; № = Number; RCT = Randomized controlled trials; RR = Risk ratio; SAVR = Surgical aortic valve replacement, TAVI = Transcatheter-aoritc valve replacment

a. Small number of events

b. Effect estimates crosses thresholds for appreciable benefits or harms

**Table B. Summary of findings regarding mortality TAVI versus MT**

| **Certainty assessment** | | | | | | | **№ of patients** | | **Effect** | | **Certainty** |  |
| --- | --- | --- | --- | --- | --- | --- | --- | --- | --- | --- | --- | --- |
| **№ of studies** | **Study design** | **Risk of bias** | **Inconsistency** | **Indirectness** | **Imprecision** | **Other considerations** | **TAVI** | **MT** | **Relative (95% CI)** | **Absolute (95% CI)** |  |  |
| **Mortality 30 days** | | | | | | | | | | | | |
| 1 | RCTs | not serious | not serious | not serious | very serious ^a^ | none | 9/179 (5.0%) | 5/179 (2.8%) | **RR 1.80** (0.62 to 5.27) | **22 more per 1.000** (from 11 fewer to 119 more) | ⨁⨁◯◯ LOW |  |
| **Mortality 30 days** | | | | | | | | | | | | |
| 1 | Propensity-matched studies | not serious | not serious | not serious | very serious ^a^ | none | 16/135 (11.9%) | 10/135 (7.4%) | **RR 1.60** (0.75 to 3.40) | **44 more per 1.000** (from 19 fewer to 178 more) | ⨁◯◯◯ VERY LOW |  |
| **Mortality 1 year** | | | | | | | | | | | | |
| 1 | RCTs | not serious | not serious | not serious | serious ^a,b,c^ | none | 55/179 (30.7%) | 89/179 (49.7%) | **RR 0.62** (0.47 to 0.81) | **189 fewer per 1.000** (from 94 fewer to 264 fewer) | ⨁⨁⨁◯ MODERATE |  |
| **Mortality 1 year** | | | | | | | | | | | | |
| 1 | Propensity-matched studies | not serious | not serious | not serious | serious ^b^ | none | 28/135 (20.7%) | 69/135 (51.1%) | **RR 0.41** (0.28 to 0.59) | **302 fewer per 1.000** (from 210 fewer to 368 fewer) | ⨁◯◯◯ VERY LOW |  |

**Abbreviations:** CI = Confidence interval; MT = Medical therapy; № = Number; RCT = Randomized controlled trials; RR = Relative risk, TAVI = Transcatheter-aoritc valve replacment

a. Small number of events

b. Sample size does not meet optimal information size

c. Consistent with observational study
