# Supplementary material for: Comparison of transcatheter aortic valve implantation with other approaches to treat aortic valve stenosis: a systematic review and meta-analysis
Source: Syst Rev. 2019 Feb 5;8:44. doi: 10.1186/s13643-019-0954-3 (PMC6362570; doi:10.1186/s13643-019-0954-3)
Supplement: Supplementary file 7 — Subgroup analysis. (DOCX 35 kb) [file 13643_2019_954_MOESM7_ESM.docx]

**Additional file 7. Subgroup analysis**

**Figure A. Forest plot for 30 days mortality in studies with high and low or intermediate risk patient populations**

**Abbreviations:** CI = Confidence interval, D+L = DerSimonian and Laird method, M-H = Mantel-Haenszel method, NOTION = Nordic Aortic Valve Intervention, OBSERVANT = Observational Study of Effectiveness of SAVR-TAVR Procedures for Severe Aortic Stenosis Treatment, PARTNER = Placement of Aortic Transcatheter Valves, RCT = Randomized controlled trial, RR = Relative Risk, SAVR = Surgical aortic valve replacement, SURTAVI = Surgical Replacement and Transcatheter Aortic Valve Implantation, TAVI = Transcatheter-aoritc valve replacment, US = United States

**Figure B. Forest plot for one year mortality in studies with high and low or intermediate risk patient populations**

**Abbreviations:** CI = Confidence interval, D+L = DerSimonian and Laird method, M-H = Mantel-Haenszel method, NOTION = Nordic Aortic Valve Intervention, OBSERVANT = Observational Study of Effectiveness of SAVR-TAVR Procedures for Severe Aortic Stenosis Treatment, PARTNER = Placement of Aortic Transcatheter Valves, RCT = Randomized controlled trial, RR = Relative Risk, SAVR = Surgical aortic valve replacement, SURTAVI = Surgical Replacement and Transcatheter Aortic Valve Implantation, TAVI = Transcatheter-aoritc valve replacment, US = United States
